# Discovery of Guanidine Derivatives from *Buthus martensii* Karsch with Metal-Binding and Cholinesterase Inhibition Properties

**DOI:** 10.3390/molecules26216737

**Published:** 2021-11-08

**Authors:** Yu-Ming Liu, Jing-Jing Fan, Li-Ning Wang

**Affiliations:** 1 Department of Pharmacy Engineering, Tianjin University of Technology, Tianjin 300384, China; lgdx19407@163.com; 2 Tianjin Key Laboratory of Drug Targeting and Bioimaging, Tianjin University of Technology, Tianjin 300384, China; 3 College of Traditional Chinese Medicine, Tianjin University of Traditional Chinese Medicine, Tianjin 300193, China; liningwang@aliyun.com

**Keywords:** *Buthus martensii* Karsch, cholinesterase inhibitor, guanidine-type alkaloid, metal-binding, molecular docking, Alzheimer’s disease (AD), natural products

## Abstract

Two rare guanidine-type alkaloids, Buthutin A (**1**) and Buthutin B (**2**), along with two other compounds (**3**, **4**), were isolated from *Buthus martensii* Karsch, and determined using extensive spectroscopic data analysis and high resolution-mass spectrometry. Compound **1** showed the most potent inhibition on AChE and BChE with IC_50_ values of 7.83 ± 0.06 and 47.44 ± 0.95 μM, respectively. Kinetic characterization of compound **1** confirmed a mixed-type of AChE inhibition mechanism in accordance with the docking results, which shows its interaction with both catalytic active (CAS) and peripheral anionic (PAS) sites. The specific binding of compound **1** to PAS domain of AChE was also confirmed experimentally. Moreover, compounds **1** and **3** exhibited satisfactory biometal binding abilities toward Cu^2+^, Fe^2+^, Zn^2+^ and Al^3+^ ions. These results provide a new evidence for further development and utilization of *B. martensii* in health and pharmaceutical products.

## 1. Introduction

Alzheimer’s disease (AD) is a neurodegenerative disorder characterized by progressive cognitive impairment and memory loss. With the acceleration of the aging process of the world population, the incidence of AD increases year by year, and it is estimated that the number of AD patients worldwide will exceed 100 million by 2050.

Although AD pathogenesis has not been fully identified, it is confirmed to be due to the deficit of acetylcholine. Acetylcholinesterase (AChE) and butyrylcholinesterase (BChE) can catalyze the hydrolysis of acetylcholine. Previous structural studies [1,2] have been shown to characterize the overall structure of AChE into several subsites: the active site (CAS) consisting of the catalytic triad, anionic subsite and acyl binding pocket, and the peripheral anionic site (PAS) at the mouth of the gorge leading to the active site. AChE inhibitors may inhibit AChE via a competitive mechanism, by interacting with CAS of the enzyme, via a non-competitive mechanism, by binding with the peripheral anionic site (PAS), or via both mechanisms, by exerting a dual binding AChE inhibition [2]. Among the existing medications, galantamine is a strictly competitive inhibitor of AChE, while donepezil is a mixed competitive/noncompetitive inhibitor that interacts with CAS as well as with PAS of the AChE [3]. Compared with AChE, BChE plays a supportive role in the cholinergic neurotransmission. However, during the progression of AD, level of AChE in the patient brain decreases while the level and activity of BChE significantly increases. Potent BChE inhibitors may provide greater efficacy in late AD when BChE activity becomes dominant [4]. Another advantage of BChE is that, unlike AChE, it is not expressed in the peripheral and parasympathetic autonomous nervous systems, and then inhibiting BChE may not cause the adverse side-effects of AChE specific inhibitors. Therefore, the synergistic inhibition of both AChE and BChE, like rivastigmine, may be one more valuable approach to positively improve the course of AD [5].

Furthermore, the recent literature has shown that the abnormally high levels of metal ions in the brain promote the formation of Aβ plaques and catalyze the production of reactive oxygen species (ROS), which further elicit oxidative stress contributing to the AD pathogenesis [6]. Therefore, metal-binding agents are useful materials for the treatment of AD due to their beneficial effects in prevention of oxidative damage caused by free radicals [7]. Reportedly, a large proportion of active compounds and medicines currently used for central nervous system disorders are of natural origin or are modified from such compounds [8,9]. Based on the above considerations, researchers are seeking new AChE and BChE inhibitors with the multifunctional characteristics from edible and natural sources.

To date, it is well-known that alkaloidal compounds of natural origin are a great source of cholinesterase inhibitors. Scorpions are one of the oldest known groups of arthropods on earth, and over 2400 scorpion species are now widely distributed on all continents except Antarctica. Two scorpion species have been reported to have an inhibitory effect on acetylcholinesterase activity [10,11], and several alkaloids [12,13,14] have been obtained from scorpions. Consequently, the tracing of new scorpion alkaloids piqued our interest due to their possible applications for either finding or improving treatments against AD. The scorpion *Buthus martensii* Karsch is widely distributed in China, and has long been used as a tonic food for human health benefits, such as scorpion wine and fried foods for thousands of years. Recently, it has been developed as a kind of canned foods with its perfect protein and unique flavor [14,15,16]. As a Chinese traditional medicine, it is also recommended for treating convulsion, epilepsy, apoplexy, facial paralysis, hemiplegia, rheumatism and relieving pain [17,18]. Nowadays, a few small molecules including two alkaloids were investigated from the *B. martensii* Karsch [13,14,19]. As part of our ongoing investigations on the cholinesterases inhibition by natural products, we now describe the detailed bioactivities and inhibition kinetics of active alkaloids from the whole body of *B. martensii* Karsch.

## 2. Results

**Figure 1:**
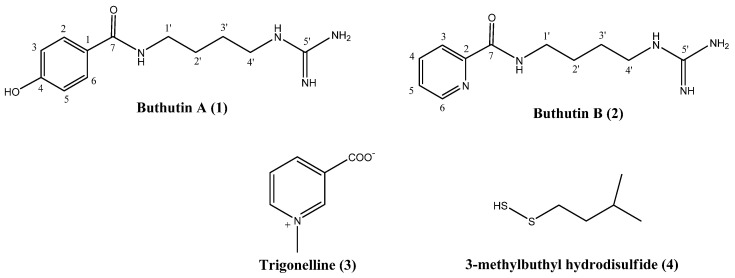
Chemical structures of compounds (**1**–**4**) from *B. martensii*.

The 85% methanolic extract of *B. martensii* was fractioned with ethyl acetate. The aqueous fraction was chromatographed on silica gel, ODS, and Sephadex LH-20 to obtain two novel compounds (**1** and **2**) designated as Buthutin A and Buthutin B, respectively, as well as the known compounds trigonelline (**3**) and 3-methylbuthyl hydrodisulfide (**4**) (Figure 1).

### 2.1. Structural Elucidation of Obtained Compounds

**Figure 2:**
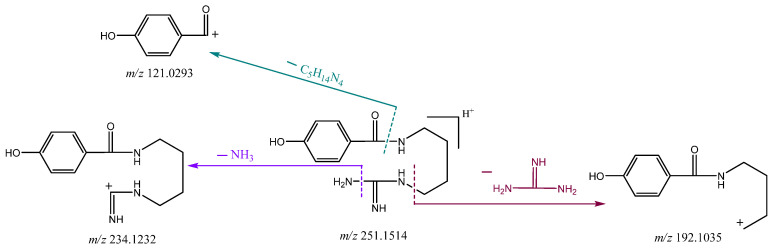
The proposed fragmentation pathways of compound **1**.

Compound **1** was isolated as a light yellowish solid, and was positive for Dragendorff reagent. Its molecular formula was established as C_12_H_18_N_4_O_2_ from a pseudmolecular ion peak at *m/z* 251.1514 [M+H]^+^ (calcd for C_12_H_19_N_4_O_2_, 251.1508) in the HR-ESI-MS (Figure S1), indicating that the molecule has six degrees of unsaturation. Based on the representative MS^+2^ spectrum (Figure S2) of compound **1**, the proposed fragmentation pathways of compound **1** are shown in Figure 2. As a result, the fragmentation of the molecular ion of compound **1** at *m/z* 251.1514 led to the predominant product ion at *m/z* 121.0293, arising from cleavage of the amide linkage. The ion at *m/z* 234.1232 could be produced by loss of an ammonia molecular. In addition, the ion at *m/z* 192.1035 could be derived from terminal loss of the neutral guanidine molecule.

**Figure 3:**
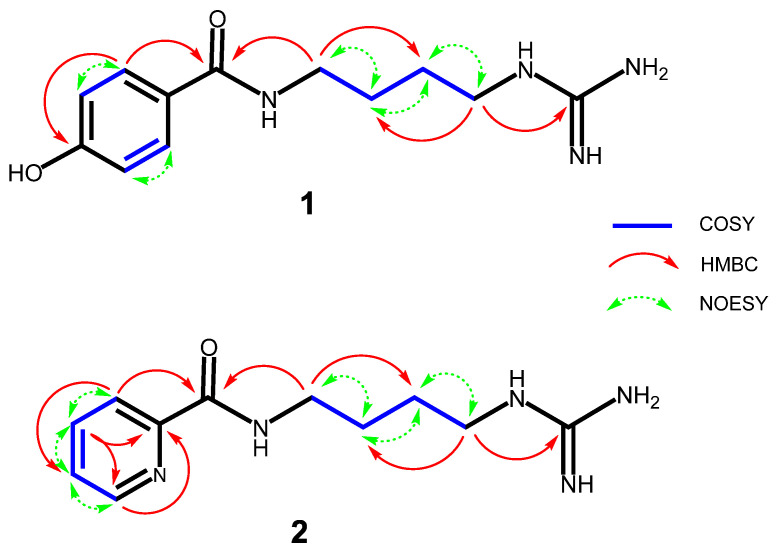
Selected 2D NMR correlation, including key ^1^H−^1^H COSY, HMBC, and NOESY, of **1** and **2**.

In accordance with the molecular formula, 12 carbon signals were resolved in ^13^C NMR spectra (Figure S4) of **1**, with aid of the DEPT (Figure S5) and HSQC (Figure S7) experiments, assignable to four sp^3^ methylenes, four protonated sp^2^ carbons, and four non-protonated sp^2^ carbons, where the downfield signal at δ 170.4 was assigned to carbonyl carbon. The ^1^H NMR spectrum (Figure S3) of **1** revealed resonances of a typical AA′XX′ spin system at δ_H_ 7.71 (H-2/6) and 6.87 (H-3/5), attributable to a 1,4-disubstituted phenyl moiety, and combination analyses of the typical chemical shift of the quaternary carbon at δ_C_ 161.2 (C-4) demonstrated that position C-4 was substituted by a hydroxyl group. The HMBC (Figure S8) correlations from H-2,6 (δ_H_ 7.71) to C-7 (δ_C_ 170.4) and from H-2,6 (δ_H_ 7.71) to C-4 (δ_C_ 161.2) confirmed the presence of a *p*-hydroxylbenzoyl substructure (Figure 3). Moreover, its ^1^H-NMR spectrum also exhibited the characteristic signals for a n-butyl unit (δ_H_ 3.38 (2H, t, *J* = 6.2 Hz, H-1′), 1.65 (2H, m, H-2′), 1.64 (2H, m, H-3′), 3.22 (2H, t, *J* = 6.2 Hz, H-4′)), which was further confirmed by successive ^1^H-^1^H COSY (Figure S6) connections from 1′-CH_2_ (δ_H_ 3.38) to 4′-CH_2_ (δ_H_ 3.22) (Figure 3). The methylene protons at δ_H_ 3.22 and δ_H_ 3.38 appeared as low field values due to the adjacent nitrogen atoms. The remaining elements comprised one specific quaternary carbon (δ_C_ 158.2) [20] and three nitrogen atoms representing a guanidine group. The HMBC correlation between δ_H_ 3.38 (H-1′) and δc 170.4 (C-7) was observed, indicating that the *n*-1,4-butyldiamine chain was directly attached to the carbonyl carbon at C-7; the HMBC correlation from H-4′ (δ_H_ 3.22, t, *J* = 6.2 Hz) to C-5′ (δ_C_ 158.2) confirmed that C-4′ (δc 42.1) of the *n*-1,4-butyldiamine chain was directly attached to the guanidine group (Figure 3). On the base of further analyses of its DEPT, ^1^H–^1^H COSY, HSQC, HMBC and NOESY (Figure S9) spectra, compound **1** was identified as *N*-(4-guanidinobutyl)-4-hydroxybenzamide (Figure 1) and was given the trivial name Buthutin A. Although it was previously reported in a synthetic study [21], this is the first report of its occurrence in nature.

Compound **2** was afforded to be a yellowish white solid and was positive for Dragendorff reagent. HR-ESI-MS (Figure S10) of **2** displayed the pseudmolecular ion peak at *m*/*z* 236.1524 [M+H]^+^ (calcd for C_11_H_18_N_5_O, 236.1511) corresponding to the molecular formula C_11_H_17_N_5_O with six degrees of unsaturation, which was CHO less than compound **1** instead of the addition of one nitrogen atom. The ^13^C NMR data (Figure S12) showed 11 carbon signals, classified by the HSQC (Figure S15) and DEPT (Figure S13) spectra, including one amide (δ_C_ 167.1), one guanidine group (δ_C_ 158.8), one non-protonated sp^2^ carbon (δ_C_ 151.1), four protonated sp^2^ carbons (δ_C_ 123.2, 139.0, 127.9, and 150.0), four methylene carbons (δ_C_ 39.9, 27.9, 27.3, and 42.3). NMR data comparison indicated that the signals assigned to *n*-butyl and guanidine units in **2** were the same as those of compound **1**, indicating the presence of a *n*-butyl-guanidine moiety in **2**. The HMBC (Figure S16) correlation of H-4′ (δ_H_ 3.26, t, *J* = 6.9 Hz) with C-5′ (δ_C_ 158.8) further confirmed that the *n*-butyl unit was linked to guanidine group via the C-4′—N—C-5′ bond (Figure 3). In the other regions of its ^1^H NMR spectrum (Figure S11), one set of characteristic ABMX system aromatic protons at δ_H_ 8.11 (1H, d, *J* = 7.7 Hz, H-3), 7.98 (1H, t, *J* = 7.7 Hz, H-4), 7.57 (1H, dd-like, *J* = 4.4, 7.7 Hz, H-5) and 8.66 (1H, d, *J* = 4.4 Hz, H-6) was revealed. Additionally, the HMBC correlations from H-3 (δ_H_ 8.11, d, *J* = 7.7 Hz) to C-7 (δ_C_ 167.1), together with the 1H–1H COSY (Figure S14) and NOESY (Figure S17) correlations of H-3↔H-4↔H-5↔H-6, established the picolinamide derivative moiety of **2** (Figure 3), which would also coincide with its molecular formula (C_11_H_17_N_5_O) as a whole. The HMBC correlation between δ_H_ 3.49 (H-1′) and δ_C_ 167.1 (C-7) was observed (Figure 3), indicated that the n-butyl chain was attached to the amide carbon at C-7 via the C-1′—N—C-7 bond. Based on above analysis, compound **2** was found to be *N*-(4-guanidinobutyl)picolinamide (Figure 1) and was given the trivial name Buthutin B. This compound is a new substance as no literature data have been reported before.

Compounds **3**–**4** were identified as *N*-methylnicotinic acid, i.e., trigonelline (**3**) [22] and 3-methylbuthyl hydrodisulfide (**4**) [23] by comparison of their NMR (Figures S19–S24, S26 and S27) and MS (Figures S18 and S25) spectroscopic data with those reported in the literature. Additionally, compound **4** is only the second report of its occurrence in nature.

### 2.2. Cholinesterase Inhibition Activities

Inhibitory activities on AChE and BChE in comparison to the reference compound galanthamine were determined. The crude extract, ethyl acetate soluble fraction, and aqueous fraction of *B. martensii* Karsch were tested for AChE inhibitory activity, and the aqueous fraction possessed particular inhibitory activity with its IC_50_ value of 56.8 ± 3.72 μg/mL. Accordingly, we screened the cholinesterase inhibitory activities of the isolated compounds from this fraction. The IC_50_ values of tested compounds and their selectivity indexes (SI) for AChE over BChE are listed in Table 1. In the AChE assay, compound **1** displayed the most potent inhibitory activity with IC_50_ value of 7.83 ± 0.06 μM, and compounds **2** and **4** showed moderate inhibitory activity, with IC_50_ values of 61.45 ± 2.34 and 40.93 ± 3.21 μM, respectively, while compound **3** had a weaker inhibitory effect. In terms of inhibitory activity against BChE, compounds **1**–**4** showed higher IC_50_ values than those for AChE inhibition with selectivity index ranging from 1.99 to 6.05, indicating that all compounds **1**–**4** acted as selective AChE inhibitors. The significantly high inhibition of compound **1** than compound **2** indicated that substitution of the hydroxyl group at C-4 and electron deficient pyridine ring could be the indelible factors could be an indelible factor for AChE and BChE inhibitory activities.

### 2.3. Propidium Iodide Displacement Assay

Propidium iodide is a known specific inhibitor of peripheral anionic site (PAS) of AChE. The binding abilities of compounds **1**–**4** to PAS site was determined by competitively displacing the propidium iodide. The results obtained in Table 1 showed that compounds **1** (18.29 ± 0.53%) and **2** (17.95 ± 0.98%) highly displaced propidium iodide from the propidium iodide–AChE enzyme complex, which are comparable with those of donepezil (18.50 ± 1.13%). Compounds **3** and **4** appeared to have considerably less capable in displacing the propidium iodide from AChE. These results indicated that compounds **1** and **2** could efficiently bind to the PAS site of AChE.

### 2.4. Enzyme Kinetic Analyses against AChE

To gain further insight into the mechanism of AChE enzyme inhibition and to understand the dual-binding site character, enzyme kinetic analyses were performed on active compounds **1** and **2**. The results from Figure S29 exhibited that compounds **1** and **2** are reversible inhibitors, as in the presence of different concentrations of compounds, plots of the initial velocity versus enzyme concentration gave a series of straight lines, all of which passed through the origin.

**Figure 4:**
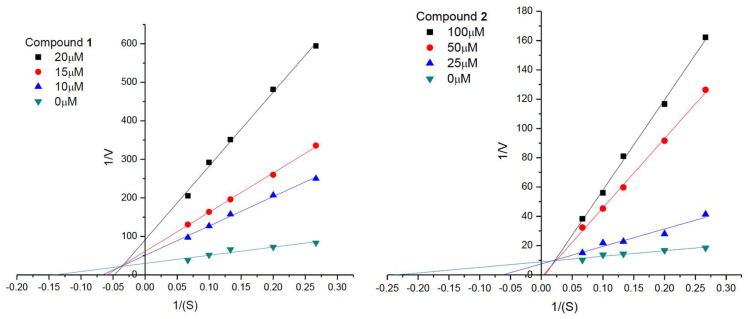
Lineweaver–Burk plots for the inhibition of AChE by compounds **1** and **2**.

**Figure 5:**
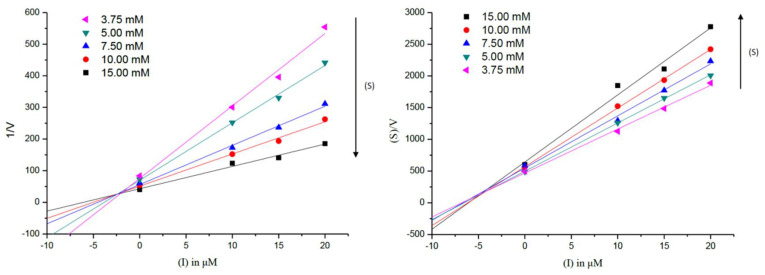
Dixon (**left**) and Cornish–Bowden (**right**) plots for most active AChE inhibitor **1**.

The kinetic characterization of compounds **1** and **2** against AChE was also carried out by measuring the enzyme’s activity at different concentrations of substrate. The results in Figure 4 showed that compounds **1** and **2** had a family of straight lines with different slopes but they intercepted one another in the first and second quadrant, respectively. All of these lines had no intersection on the horizontal or vertical axis, indicating that compounds **1** and **2** cause a mixed type of inhibition, thus supporting the dual binding character of compounds **1** and **2** that bind, in all likelihood, to both catalytic active site (CAS) and PAS of the enzyme. Dixon and Cornish–Bowden plots (Figure 5) for compound **1** showed inhibition constants *K*_i,c_ = 2.64 ± 0.35 μM for the competitive (c) component, and *K*_i,nc_ = 5.66 ± 0.82 μM for the non-competitive (nc) component.

### 2.5. Metal-Binding Studies

**Figure 6:**
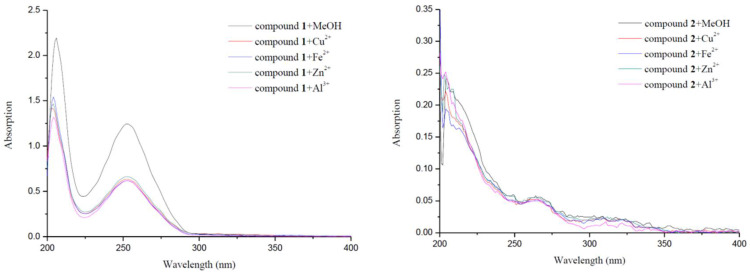

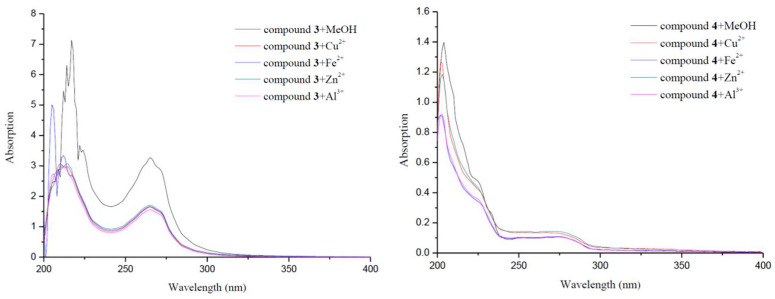
UV spectra of compounds **1**–**4** alone or in the presence of CuCl_2_, FeCl_2_, ZnCl_2_ or AlCl_3_.

The ability of the synthesized compounds in binding metals would be an added advantage in the treatment of AD. Herein, compounds **1**–**4** were studied for their binding abilities toward Cu^2+^, Fe^2+^, Zn^2+^ and Al^3+^, using UV spectrophotometer with wavelengths ranging from 200 nm to 400 nm. As shown in Figure 6, the spectra of compounds **1** and **3** were significantly changed upon the addition of CuCl_2_, FeCl_2_, ZnCl_2_ and AlCl_3_. The dramatic decreases in absorbance indicated the possible interactions between these biometals and compounds **1** and **3**. The potent metal binding ability of compounds **1** and **3** could be due to the contribution of hydroxyl group and the donation of carboxyl anion, respectively.

### 2.6. Molecular Docking Studies

**Figure 7:**
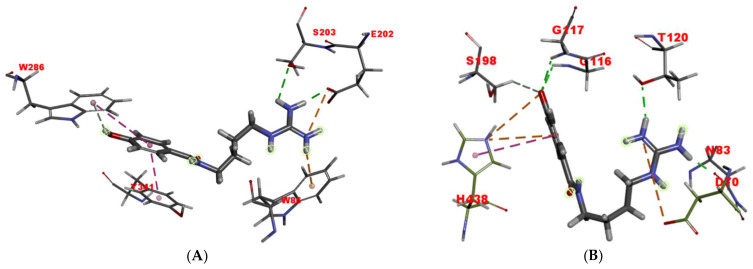
Docking models of compound **1** with AChE (**A**) and BChE (**B**).

To investigate the binding pattern of compound **1** with AChE, molecular docking studies were performed using Discovery Studio (Figure 7A). In the CAS, a conventional hydrogen bond was formed between the guanidine group and hydroxyl of Ser203. The guanidine group and Trp86 also interacted with π–cation. In addition, the carboxyl of Glu202 residue, which plays an important role in molecular recognition and binding of specific ligands to the catalytic triad [24], interacted with the guanidine group by a salt bridge and a conventional hydrogen bond. For another, the phenyl moiety of compound **1** was located at the PAS, and formed two π–π stacked interactions with Trp286 and Tyr341. Its hydroxyl group also interacted with the benzene ring of Trp286 through a π-donor hydrogen bond. This interaction study demonstrated that compound **1** was strongly bound to both the binding sites CAS and PAS of AChE, which showed a consistent inhibitory pattern on AChE to what revealed by its kinetic study and propidium displacement test. As the PAS of AChE is involved in an increased Aβ aggregation rate, dual interaction with two binding sites (CAS and PAS) would be especially advantageous for slowing the progression of AD [24,25].

The interaction of compound **1** with BChE was also carried out. As seen in Figure 7B, compound **1** was bound to the residues Gly117 and Gly116 from the oxyanion hole (OAH), Ser198 and His438 from CAS, Asp70 from PAS, and additionally to Thr120 and Asn83 residues, via two charge interactions, one π–cation interaction, one π–π T-shaped interaction, four conventional hydrogen bonds, and one carbon hydrogen bond. Compared to its extended conformation bound to AChE, compound **1** exhibited a somewhat U-shaped conformation, which might partly explain its lower inhibitory potency.

## 3. Materials and Methods

### 3.1. General Experimental Procedures

A Waters Acquity UPLC Class I/Xevo G2Q-Tof mass spectrometer (Milford, MA, USA) was used to obtain the high-resolution ESI-MS (HR-ESI-MS) data. ^1^H and ^13^C NMR spectra were acquired on a Bruker Avance III 400 spectrometer spectrometer (Karlsruhe, Germany) with TMS as an internal standard. ^1^H-^1^H DQF-COSY, NOESY, TOCSY, HSQC and HMBC spectra were recorded using standard Bruker programs spectrometer (Topspin 3.5pl6, Karlsruhe, Germany). Middle pressure liquid chromatography (MPLC) was carried out on a BUCHI apparatus spectrometer (BUCHI LABORTECHNIK AG, Flawil, Switzerland) equipped with a C-605 pump and open chromatographic columns of different specifications (without detector). Thin layer chromatography (TLC) analysis was performed on alumina plates coated with silica gel 60 and silica gel 60 RP-18 F_254_ (Merck) spectrometer (Darmstadt, Germany). Chromatographic separation was performed on self-packed columns with silica gel (Anhui Liangchen Silicon Material Co. Ltd., China) and reversed-phase silica gel (ODS-A 12 nm S-50 μm YMC Co., Kyoto, Japan). AChE (EC 3.1.1.7, from electric eel), BChE (EC 3.1.1.8, from equine serum), 5,5′-dithiobis(2-nitrobenzoic acid) (DTNB), acetylthiocholine iodide (ATCI), and butyrylthiocholine iodide (BTCI) were purchased from Sigma-Aldrich (St. Louis, MO, USA).

### 3.2. Arthropod Material

Dried body of *B. martensii* Karsch were purchased from a Chinese medicine store in Anguo, Hebei of China, and identified by Prof. Yu-Ming Liu. A voucher specimen (HB-17-1203) was deposited at the Department of Pharmacy Engineering, Tianjin University of Technology.

### 3.3. Extraction and Isolation

*B. martensii* Karsch (2 kg) were crushed and exhaustively extracted with 85% methanol for three times (each for 3 h) under reflux. The obtained crude extract (337 g) was suspended in distilled water and partitioned with ethyl acetate to obtain the organic fraction and aqueous fraction. The aqueous layer (110 g) was subjected to normal silica gel column chromatography (CC) (9 × 60 cm, 300–400 mesh using a gradient system of CH_2_Cl_2_-MeOH (20:1, 15:1, 10:1, 5:1, 1:1 *v*/*v*; 3.5 L each) to correspondingly obtain five fractions (A–E). Fraction D and E both showed more potent inhibition (IC_50_ < 50 μg/mL) on AChE than any one of fraction A–C. Then, fraction D was separated into four subfractions (Fr.D1–4) by vacuum silica gel CC (6 × 60 cm, 300–400 mesh with CH_2_Cl_2_-MeOH (15:1 *v*/*v*; 4.0 L). Fr.D3 (between 2.5 L and 3.0 L) was further loaded on ODS MPLC (26 × 460 mm) with MeOH-H_2_O (3:7 *v*/*v*; 4.5 mL/min) to yield compound **4** (9.2 mg; between 0.09 L and 0.11 L). Fraction E was chromatographed by normal silica gel CC (7 × 60 cm, 300–400 mesh) with a CH_2_Cl_2_-MeOH gradient (6:1, 3:1, 1:1 *v*/*v*; 2.0 L each) to gain six subfractions (Fr.E1–6), Then, Fr.E4 (between 3.0 L and 4.0 L) was separated over vacuum silica gel CC (5.5 × 60 cm, 300–400 mesh) using the isometric solvent system of CH_2_Cl_2_-MeOH (10:1 *v*/*v*; 1 L each) to correspondingly gain six subfractions (Fr.E4.1–E4.6). Fr.E4.5 was subjected to ODS MPLC (26 × 460 mm) using a gradient solvent system of MeOH-H_2_O (1:9, 2:8, 4:6, 5:5, 9:1 *v*/*v*; 0.27 L each; 4.5 mL/min) to yield Fr.E4.5.1–E4.5.5. Further purification of Fr.E4.5.2 was carried out using ODS MPLC (16 × 460 mm) with MeOH-H_2_O (1:4, *v*/*v*; 2.0 mL/min) to afford compound **1** (53 mg; between 0.04 L and 0.05 L), while Fr.E4.5.3 was isolated using ODS MPLC (16 × 460 mm) with MeOH-H_2_O (1:4, *v*/*v*; 2.0 mL/min) to give compound **2** (18 mg; between 0.08 L and 0.09 L). Fr.E6 (between 5.0 L and 6.0 L) was separated into five subfractions (Fr.E6.1–6.5) by normal silica gel CC (4 × 40 cm, 300–400 mesh) eluted with CH_2_Cl_2_-MeOH (2:1 *v*/*v*; 1.5 L). Fr.E6.3 (between 0.75 L and 0.90 L) was further purified by Sephadex LH-20 (2 × 60 cm, MeOH, 0.35 L) to yield compound **3** (22 mg; between 0.17 L and 0.19 L). All samples were isolated by self-packed columns, detected by TLC, and manual collection was performed. Before applying to the ODS column for MPLC, dried sample was dissolved using initial mobile phase or soluble solvents with small volume.

#### 3.3.1. *N*-(4-Guanidinobutyl)-4-hydroxybenzamide (**1**)

Light yellowish solid; Mp. 160−162 °C; HR-ESI-MS (positive mode) *m/z*: 251.1514 [M+H]^+^ (calculated for C_12_H_19_N_4_O_2_, 251.1508); HR-ESI-MS^+2^ (M′ = 251.1514 [M+H]^+^) *m/z*: 234.1232 (calculated for C_12_H_16_N_3_O_2_, 234.1243), 192.1035 (calculated for C_11_H_14_NO_2_, 192.1025), 121.0293 (calculated for C_7_H_5_O_2_, 121.0289); ^1^H NMR (400 MHz, CD_3_OD) δ: 1.64 (2H, m, H-3′), 1.65 (2H, m, H-2′), 3.22 (2H, t, *J* = 6.2 Hz, H-4′), 3.38 (2H, t, *J* = 6.2 Hz, H-1′), 6.87 and 7.71 (4H, AA′XX′, H-3/5 and H-2/6); ^13^C NMR (100 MHz, CD_3_OD) δ: 170.4 (C-7), 161.2 (C-4), 158.2 (C-5′), 130.3 (C-2 and C-6), 126.4 (C-1), 116.3 (C-3 and C-5), 42.1 (C-4′), 40.2 (C-1′), 27.5 (C-2′), 27.0 (C-3′).

#### 3.3.2. *N*-(4-Guanidinobutyl)picolinamide (**2**)

Yellowish white solid; Mp. 171−173 °C; HR-ESI-MS (positive mode) *m/z*: 236.1524 [M+H]^+^ (calculated for C_11_H_18_N_5_O, 236.1511); ^1^H NMR (400 MHz, CD_3_OD) δ: 1.69 (2H, m, H-3′), 1.71 (2H, m, H-2′), 3.26 (2H, t, *J* = 6.9 Hz, H-4′), 3.49 (2H, t, *J* = 6.5 Hz, H-1′), 7.57 (1H, dd-like, *J* = 4.4, 7.7 Hz, H-5), 7.98 (1H, t, *J* = 7.7 Hz, H-4), 8.11 (1H, d, *J* = 7.7 Hz, H-3), 8.66 (1H, d, *J* = 4.4 Hz, H-6); ^13^C NMR (100 MHz, CD_3_OD) δ: 167.1 (C-7), 158.8 (C-5′), 151.1 (C-2), 150.0 (C-6), 139.0 (C-4), 127.9 (C-5), 123.2 (C-3), 42.3 (C-4′), 39.9 (C-1′), 27.9 (C-2′), 27.3 (C-3′).

#### 3.3.3. *N*-Methylnicotinic Acid (**3**)

Mp. 218−221 °C (dec); HR-ESI-MS *m/z*: 138.0566 [M+H]^+^ (calculated for C_7_H_8_NO_2_, 138.0555); ^1^H NMR (400 MHz, CD_3_OD) δ: 4.52 (3H, s, *N*-CH_3_), 8.13 (1H, t-like, *J* = 7.3 Hz, H-5), 8.93 (1H, d, *J* = 7.9 Hz, H-4), 9.00 (1H, d, *J* = 5.8 Hz, H-6), 9.29 (1H, s, H-2); ^13^C NMR (100 MHz, CD_3_OD) δ: 167.1 (-COO^-^), 147.6 (C-2), 147.2 (C-6), 146.0 (C-4), 139.5 (C-3), 128.7 (C-5), 49.2 (*N*-CH_3_).

#### 3.3.4. 3-Methylbuthyl Hydrodisulfide (**4**)

Colorless gum; HR-ESI-MS (positive mode) *m/z*: 137.0493 [M+H]^+^ (calculated for C_5_H_13_S_2_, 137.0459); ^1^H NMR (400 MHz, DMSO-*d*_6_) δ: 0.87 (6H, d, *J* = 6.6 Hz, H-4 and H-5), 1.42 (2H, q, *J* = 8.0 Hz, H-2), 1.60 (1H, m, H-3), 2.78 (2H, t, *J* = 8.0 Hz, H-1), 5.47 (1H, s, -SSH); ^13^C NMR (100 MHz, DMSO-*d*_6_) δ: 37.7 (C-1), 36.3 (C-2), 25.4 (C-3), 22.6 (C-4 and C-5).

### 3.4. Cholinesterase Inhibitory Assay

The AChE (BChE) inhibitory activities of compounds **1**–**4** were determined by using modified Ellman’s method [26,27]. AChE (EC 3.1.1.7, from electric eel) and BChE (EC 3.1.1.8, from equine serum) were purchased from Sigma-Aldrich (St. Louis, MO, USA). Each of 96-well microtiter plates was added 140 μL phosphate buffer (0.1 mol/L, pH 8.0), 20 μL each sample (compound **1**: 0.5–100 μM for AChE and BChE; compound **2**–**4**: 1–200 μM for AChE and 10–800 μM for BChE) and 20 μL AChE (BChE) solution (0.05 U/mL). After reaction for 15 min at 25 °C, 10 μL of 10 mM DTNB and 10 μL of 7.5 mM ATCI (BTCI) were added and then incubated for 40 min at 37 °C. The absorbance of each sample was read at 412 nm by using a microplate reader (Ai) (BioTek Instruments Inc., Winooski, VT, USA). Blank groups were set up by replacing the sample solution with 20 μL phosphate buffer (Ac). All tests were performed in triplicate. The inhibition rate (%) was calculated by the following equation: (Ac − Ai)/Ac × 100%. IC_50_ values (the concentration of test compounds required to inhibit enzyme activity by 50%) were calculated with Origin 8.0 (OriginLab Corporation, Northampton, MA, USA).

### 3.5. Propidium Iodide Displacement Assay

The assay mixture of AChE (5U) was incubated with test compounds (final concentration 100 μM, 150 μL) for 6 h at 25 °C. After reaction, 50 μL Propidium iodide (1 μM) was added. After 10 min, the fluorescence intensity was observed at an excitation wavelength (λex) of 535 nm and an emission wavelength (λem) of 595 nm using a fluorescence plate reader (BioTek Instruments Inc., Winooski, VT, USA). The percentage of displacement was calculated by the following expression: 100 − (IF_i_/IF_0_ × 100), where IF_i_ and IF_0_ are the fluorescence intensities with and without the test compounds, respectively [27,28].

### 3.6. Enzyme Kinetic Analysis against AChE

In order to clarify whether inhibitors interact with the target enzyme via noncovalent bond (i.e., reversibility), the initial velocity (V) of substrate hydrolysis as measured by the change in OD_412_ over the course of 2 min (after the addition of 7.5 mM ATCI) was calculated for five different concentrations of AChE (0.025, 0.04, 0.05, 0.08, and 0.10 U/mL). After enzyme activities were performed for 2 min, the inhibition type of the enzyme were analyzed by the Lineweaver–Burk plots at five different concentrations of ATCI (3.75, 5.00, 7.50, 10.00, and 15.00 mM) [27]. Inhibition constants for AChE were determined from Dixon plot (1/*V*_i_ vs. (I)) and Cornish–Bowden transformation ((S)/*V*_i_ vs. (I)) [29].

### 3.7. Metal-Binding Studies

The chelating studies were performed on an ultraviolet–visible spectrophotometry meter (HITACHI U-3900H, Tokyo, Japan). The UV absorption spectra of the test compound alone (20 μM, final concentration) or in the presence of CuCl_2_, FeCl_2_, ZnCl_2_ and AlCl_3_ (20 μM, final concentration) were recorded with the wavelength ranging from 200 to 400 nm after incubating for 30 min in methanol at room temperature [27,30].

### 3.8. Molecular Docking Studies

#### 3.8.1. Molecular Docking of Compound **1** into AChE

The binding modes were generated by using the Discovery Studio CDOCKER software (2017R2, Accelrys, San Diego, CA, USA) [31]. The crystal structure of human AChE (hAChE) in complex with donepezil (PDB code 4EY7) was taken from the Protein Data Bank. For docking purposes, the hAChE structure was previously prepared by initially adding and orienting hydrogen atoms, as well as removing waters molecules, ions and any ligands, then its structure was protonated at pH 7.4. Parameters used include: a 10.0 Å radius sphere centered at x = −13.9849, y = −43.9747 and z = 27.8950 within the human AChE active site; heating steps and cooling steps set to 2000 and 5000, respectively, while the heating and cooling temperatures were set to 700, and 300, respectively. The cocrystallized donepezil ligand was extracted and redocked in the hAChE grid for validation of the docking parameters. The poses of the cocrystallized and redocked ligands were compared using a superposition tool, and the root-mean-square deviation (RMSD) value was found to be 0.79 Å (Figure S28), indicating that the docking methods and parameters used in this study were approximate for the AchE system.

#### 3.8.2. Molecular Docking of Compound **1** into BchE

Flexible docking was carried out using Discovery Studio 2017 R2 (Accelrys, San Diego, CA, USA) [32]. The crystal structure of BchE from Homo sapiens (PDB code 4BDS) was obtained from the Protein Data Bank. The simulation was performed as our described previously [27].

## 4. Conclusions

Two rare guanidine-type alkaloids, Buthutin A (**1**) and Buthutin B (**2**), including two known compounds (**3**, **4**), were extracted from the dried body of *B. martensii*. Trigonelline (**3**), as a vitamin B_3_ homologue, has been reported as an important ingredient of fenugreek seeds and coffee beans [33], although it had never been identified in *B. martensii* Karsch. Trigonelline (**3**) has been reported to have a wide variety of biological activities including antioxidant, anti-inflammatory, hypoglycemic, hypolipidemic, anti-tumor, antimigraine, sedative, memory-improving, and neuroprotective ones [34]. In combination with the above biological activities reported in the literature, it could be concluded that trigonelline (**3**) is necessary to maintain the functional properties of *B. martensii* Karsch.

Although guanidine containing metabolites are quite rare in nature, natural guanidine derivatives have drawn continuous attention due to their potent antimicrobial, antiproliferative, antioxidant, analgesic, and anticoagulant activities [35,36]. The guanidine compounds are also known as cholinesterase inhibitors [37,38,39]. In this work, it is the first time to discover this kind of compound from the genus *Buthus*. Buthutin A (**1**) demonstrated the most potent inhibition to AChE and BChE with IC_50_ values of 7.83 ± 0.06 μM and 47.44 ± 0.95 μM, respectively. Additionally, kinetic analysis showed that Buthutin A (**1**) was a mixed-type reversible inhibitor of AChE, simultaneously binding to the catalytic and peripheral anionic sites, which was verified by in silico docking studies. Furthermore, compounds **1** and **3** showed satisfactory metal-binding properties toward Cu^2+^, Fe^2+^, Zn^2+^ and Al^3+^ ions. Thus, the edible *B. martensii* Karsch could serve as a valuable natural source of multifunctional cholinesterase inhibitors with health benefits for potential application in functional food and medicine.

## Figures and Tables

**Table 1:** Inhibitory activity on cholinesterase and propidium iodide displacement by compounds **1–4**.

Compounds	IC_50_ ^a^ (μM)		SI ^b^	Propidium Iodide Displacement
	AChE	BChE		(%)
**1**	7.83 ± 0.06	47.44 ± 0.95	6.05	18.29 ± 0.53
**2**	61.45 ± 2.34	122.64 ± 5.21	1.99	17.95 ± 0.98
**3**	97.30 ± 4.18	441.87 ± 7.99	4.54	3.52 ± 0.21
**4**	40.93 ± 3.21	152.84 ± 7.22	3.73	4.37 ± 0.16
Galanthamine	1.17 ± 0.01	18.78 ± 1.81	16.05	
Donepezil	0.049 ± 0.004	5.536 ± 0.018	112.98	18.50 ± 1.13

^a^ IC_50_ values are at least from three independent experiments and are expressed as the means ± SD. ^b^ SI for AChE = IC_50_ BChE/IC_50_ AChE.

## Data Availability

Data are contained within the article and Supplementary Material.

## Supplementary Materials

The following are available online. Figures S1–S24. 1D-NMR, 2D-NMR, and HR-ESI-MS spectra of compounds **1**–**3**; Figures S25–S27. 1D-NMR, and HR-ESI-MS spectra of compound **4**; Figure S28. Docking validation; Figure S29. Plots of initial velocity versus enzyme concentration for the inhibition of compounds **1** and **2** on the hydrolysis activity of AChE.

## Sample Availability

Samples of the compounds are available on request from the corresponding author.
