# Supplementary material for: Discovery of Guanidine Derivatives from Buthus martensii Karsch with Metal-Binding and Cholinesterase Inhibition Properties
Source: Molecules. 2021 Nov 8;26(21):6737. doi: 10.3390/molecules26216737 (PMC8588048; doi:10.3390/molecules26216737)
Supplement: Supplementary file 1 [file molecules-26-06737-s001.zip › molecules-1407418-supplementary.pdf]

Supplementary material

Discovery of guanidine derivatives from *Buthus martensii* Karsch with metal-binding and cholinesterase inhibition properties

Yu-Ming Liu [1, 2,*], Jing-Jing Fan [1] and Li-Ning Wang [3]

[1] Department of Pharmacy Engineering, Tianjin University of Technology, Tianjin 300384, PR China

[2] Tianjin Key Laboratory of Drug Targeting and Bioimaging, Tianjin University of Technology, Tianjin 300384, PR China

[3] College of Traditional Chinese Medicine, Tianjin Univerisity of Traditional Chinese Medicine, Tianjin 300193, PR China

* *Corresponding author*. Department of Pharmacy Engineering, Tianjin University of Technology, Tianjin, 300384, PR China.
E-mail address: coumarin1968@hotmail.com (Y.-M. Liu).

Table of Contents

Figure S1. HR-ESI-MS spectrum of martensine A (**1**) 3
Figure S2. HR-ESI-MS^{+2} spectrum (M′ = 251.1514 [M+H]$^{+}$) of martensine A (**1**) 4
Figure S3. ^{1}H NMR spectrum (CD_3OD) of martensine A (**1**) 5
Figure S4. ^{13}C NMR spectrum (CD_3OD) of martensine A (**1**) 6
Figure S5. DEPT 135 spectrum of martensine A (**1**) 7
Figure S6. ^{1}H-^{1}H COSY spectrum of martensine A (**1**) 8
Figure S7. HSQC spectrum of martensine A (**1**) 9
Figure S8. HMBC spectrum of martensine A (**1**) 10
Figure S9. NOESY spectrum of martensine A (**1**) 11
Figure S10. HR-ESI-MS spectrum of martensine B (**2**) 12
Figure S11. ^{1}H NMR spectrum (CD_3OD) of martensine B (**2**) 13
Figure S12. ^{13}C NMR spectrum (CD_3OD) of martensine B (**2**) 14
Figure S13. DEPT 135 spectrum of martensine B (**2**) 15
Figure S14. ^{1}H-^{1}H COSY spectrum of martensine B (**2**) 16
Figure S15. HSQC spectrum of martensine B (**2**) 17
Figure S16. HMBC spectrum of martensine B (**2**) 18
Figure S17. NOESY spectrum of martensine B (**2**) 19
Figure S18. HR-ESI-MS spectrum of trigonelline (**3**) 20
Figure S19. ^{1}H NMR spectrum (CD_3OD) of trigonelline (**3**) 21
Figure S20. ^{13}C NMR spectrum (CD_3OD) of trigonelline (**3**) 22
Figure S21. ^{1}H-^{1}H COSY spectrum of trigonelline (**3**) 23
Figure S22. HSQC spectrum of trigonelline (**3**) 24
Figure S23. HMBC spectrum of trigonelline (**3**) 25
Figure S24. NOESY spectrum of trigonelline (**3**) 26
Figure S25. HR-ESI-MS spectrum of 3-methylbuthyl hydrodisulfide (**4**) 27
Figure S26. ^{1}H NMR spectrum (DMSO-d_6) of 3-methylbuthyl hydrodisulfide (**4**) 28
Figure S27. ^{13}C NMR spectrum (DMSO-d_6) of 3-methylbuthyl hydrodisulfide (**4**) 29
Figure S28. Docking validation 30
Figure S29. Plots of initial velocity versus enzyme concentration for the inhibition of compounds **1** and **2** on the hydrolysis activity of AChE 30

Figure S1. HR-ESI-MS spectrum of martensine A (**1**)

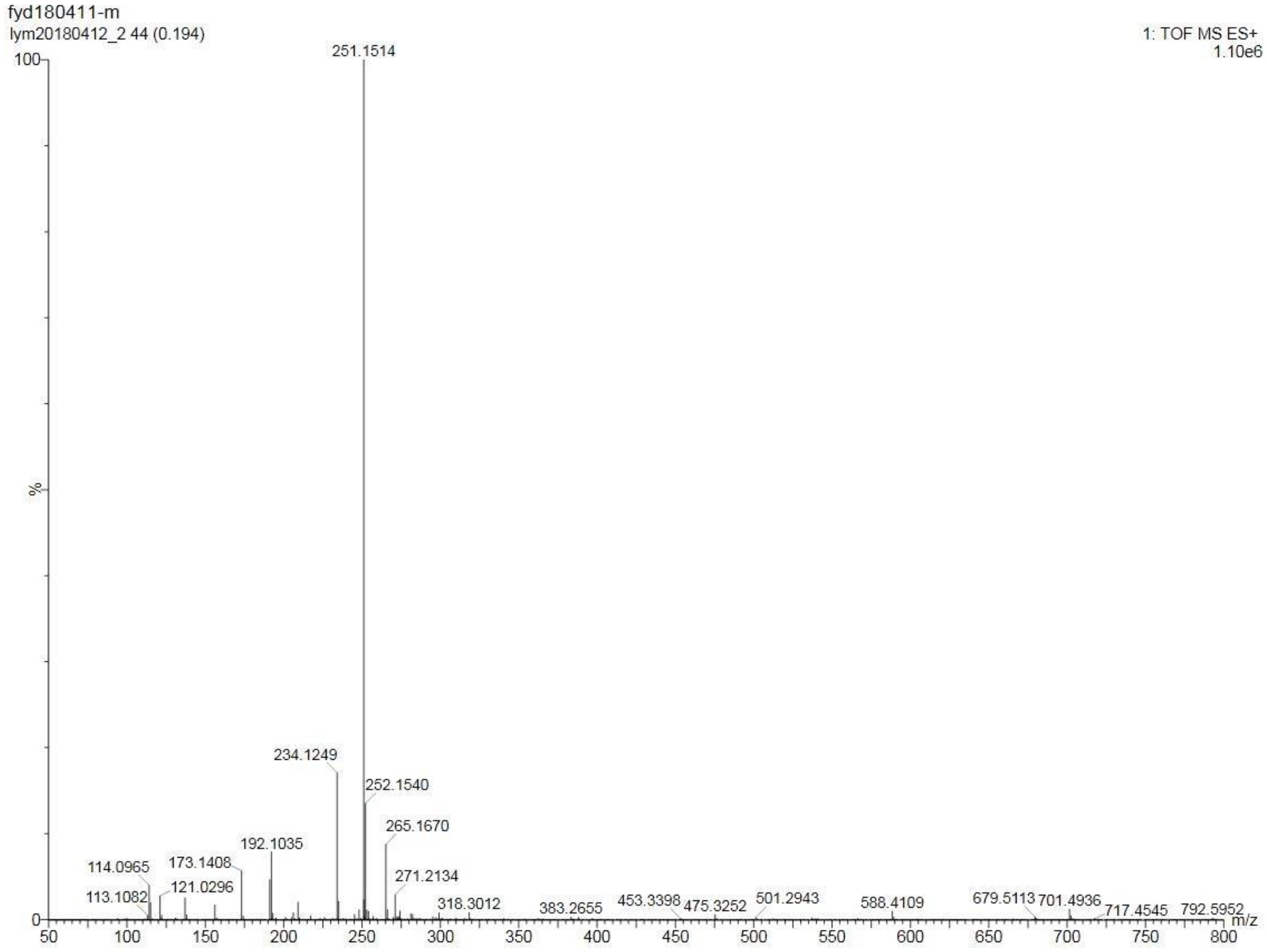

Figure S2. HR-ESI-MS^{+2} spectrum (M′ = 251.1514 [M+H]$^{+}$) of martensine A (1)

Chromatogram Report

Analysis Info

Analysis Name	D:\Data\QiaoBin\20180501-LYM-1-1.d	Acquisition Date	5/2/2018 10:41:29 AM
Method	tune_low_pos.m	Operator	BDAL@DE
Sample Name	20180428 -27- pos	Instrument / Ser#	micrOTOF-Q II 10204
Comment			

Acquisition Parameter

Source Type	ESI	Ion Polarity	Positive	Set Nebulizer	0.8 Bar
Focus	Not active	Set Capillary	4500 V	Set Dry Heater	180 °C
Scan Begin	50 m/z	Set End Plate Offset	-500 V	Set Dry Gas	6.0 l/min
Scan End	800 m/z	Set Collision Cell RF	150.0 Vpp	Set Divert Valve	Waste

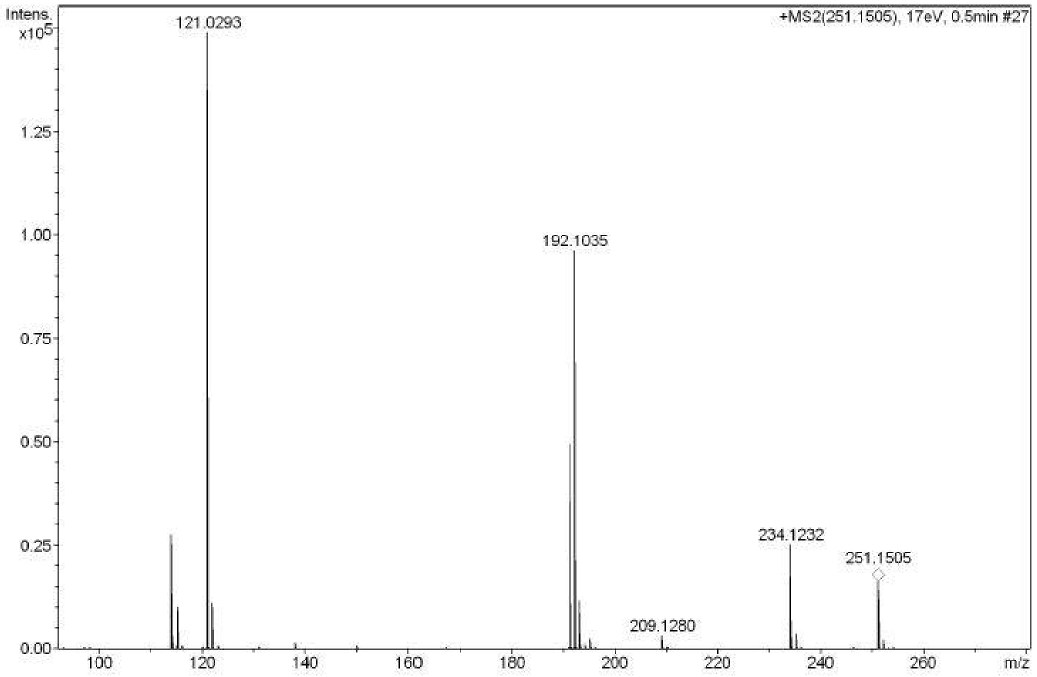

Figure S3. ^{1}H NMR spectrum (CD_3OD) of martensine A (**1**)

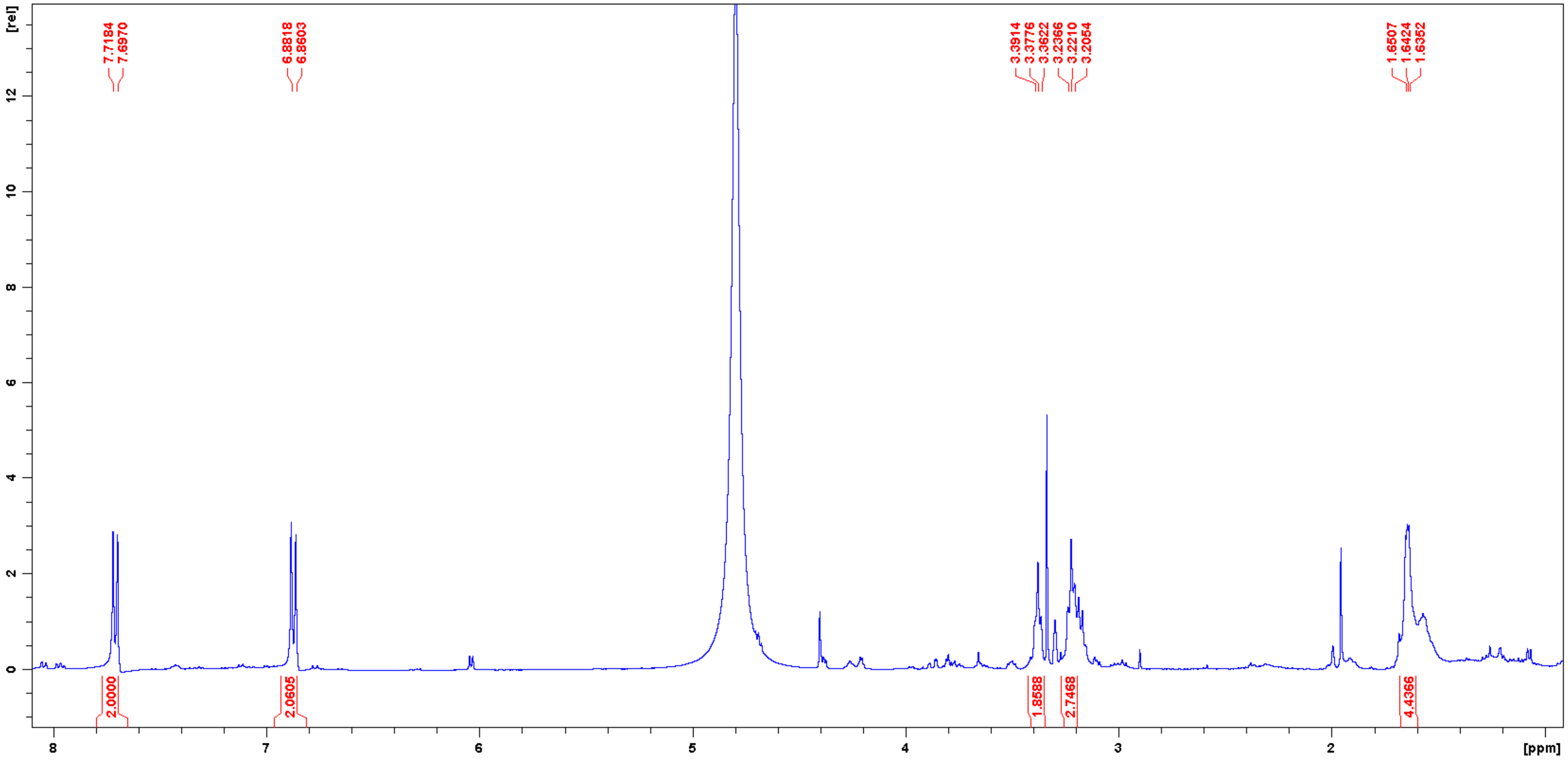

Figure S4. ^{13}C NMR spectrum (CD_3OD) of martensine A (**1**)

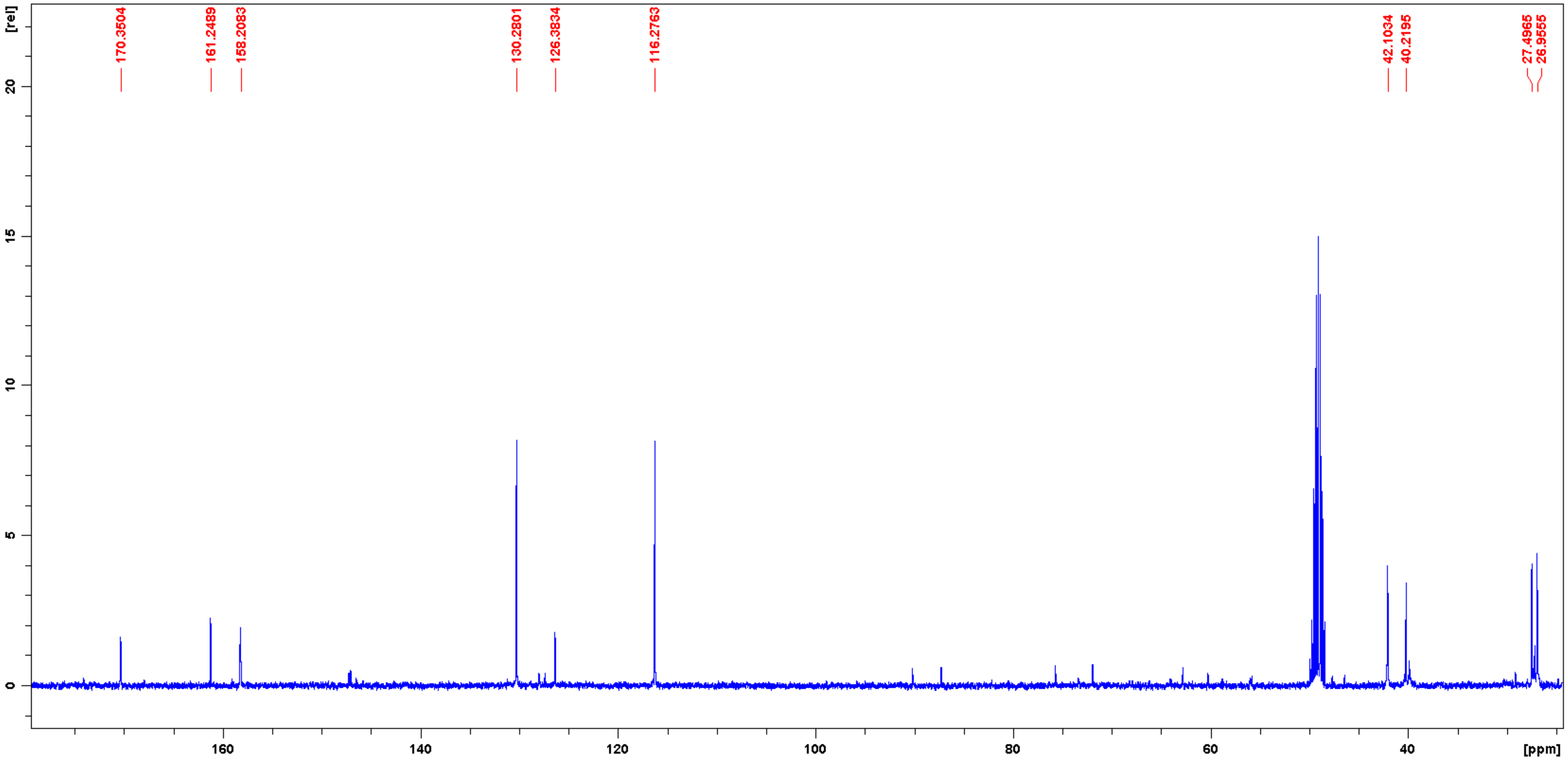

Figure S5. DEPT 135 spectrum of martensine A (**1**)

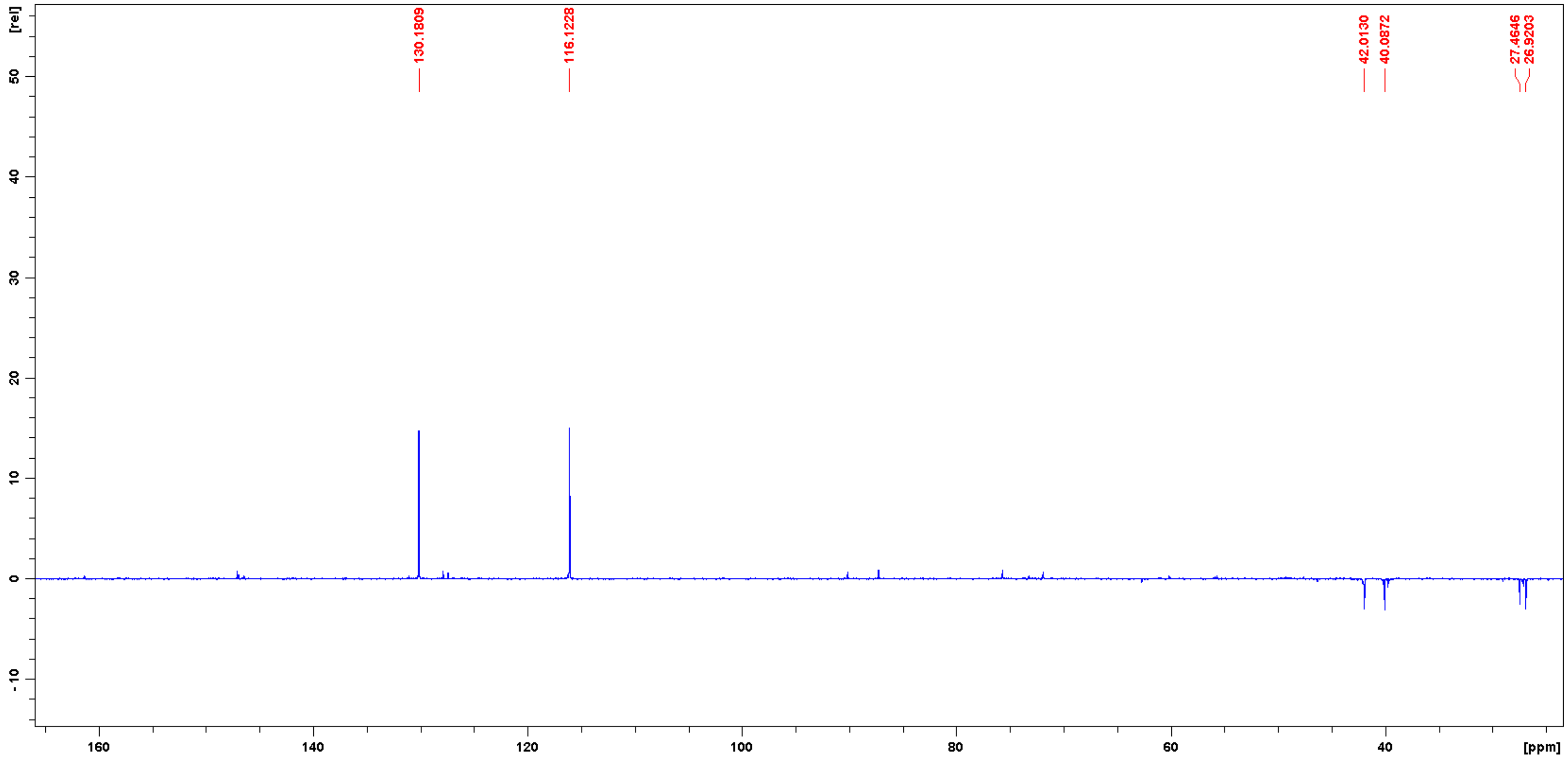

Figure S6. ^1H-^1H COSY spectrum of martensine A (**1**)

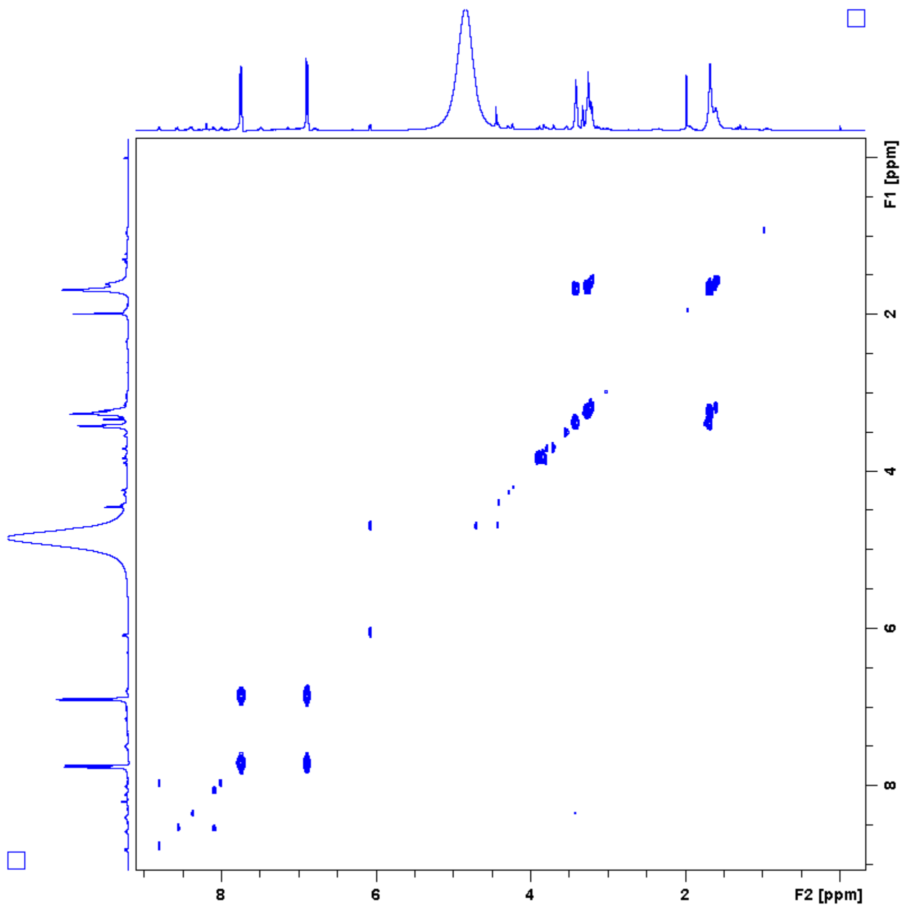

Figure S7. HSQC spectrum of martensine A (**1**)

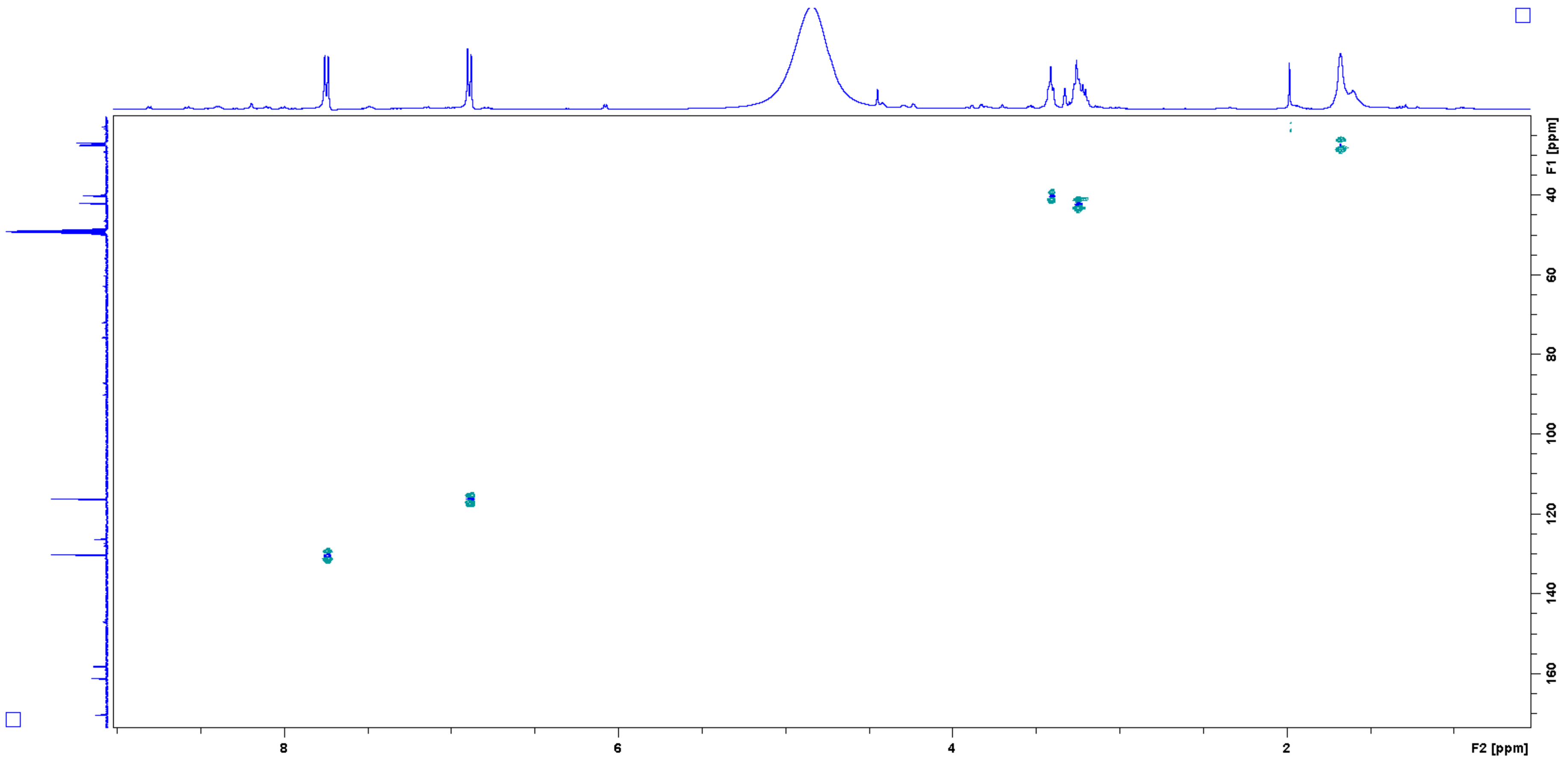

Figure S8. HMBC spectrum of martensine A (**1**)

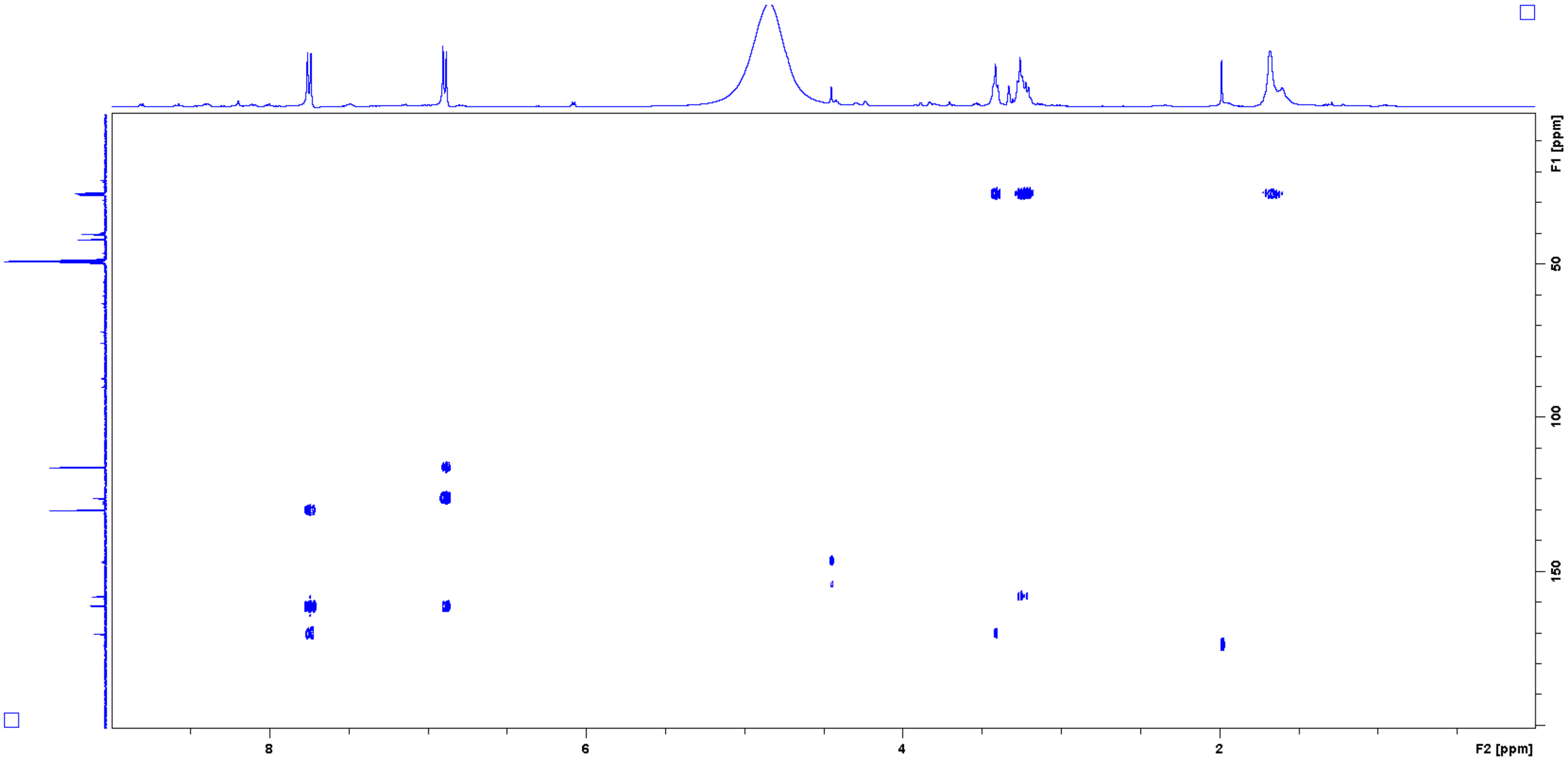

Figure S9. NOESY spectrum of martensine A (**1**)

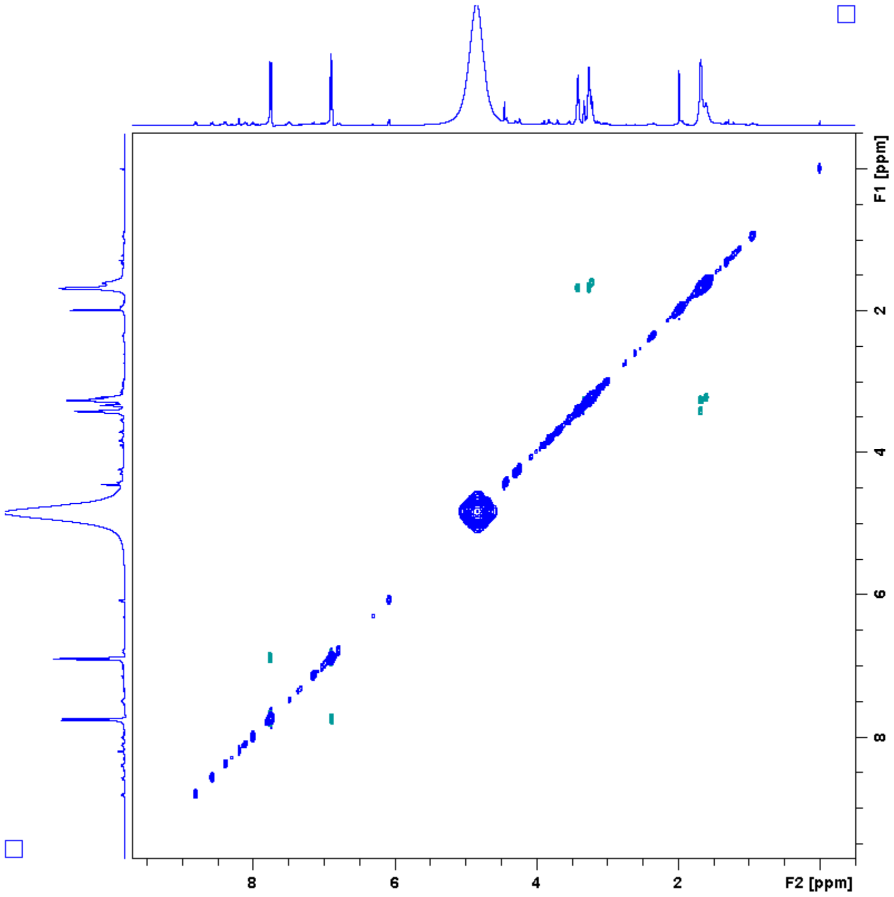

Figure S10. HR-ESI-MS spectrum of martensine B (**2**)

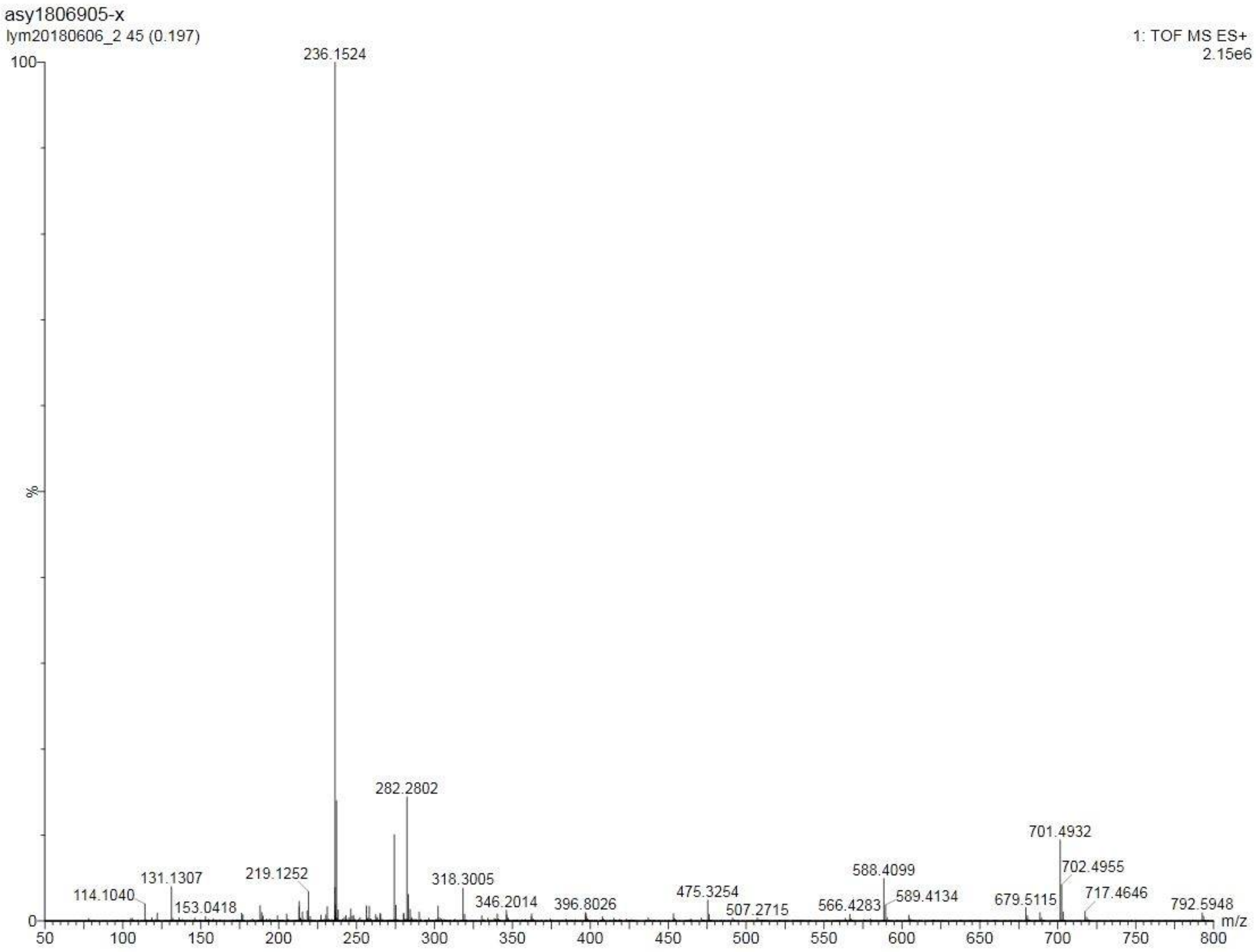

Figure S11. ^{1}H NMR spectrum (CD_3OD) of martensine B (**2**)

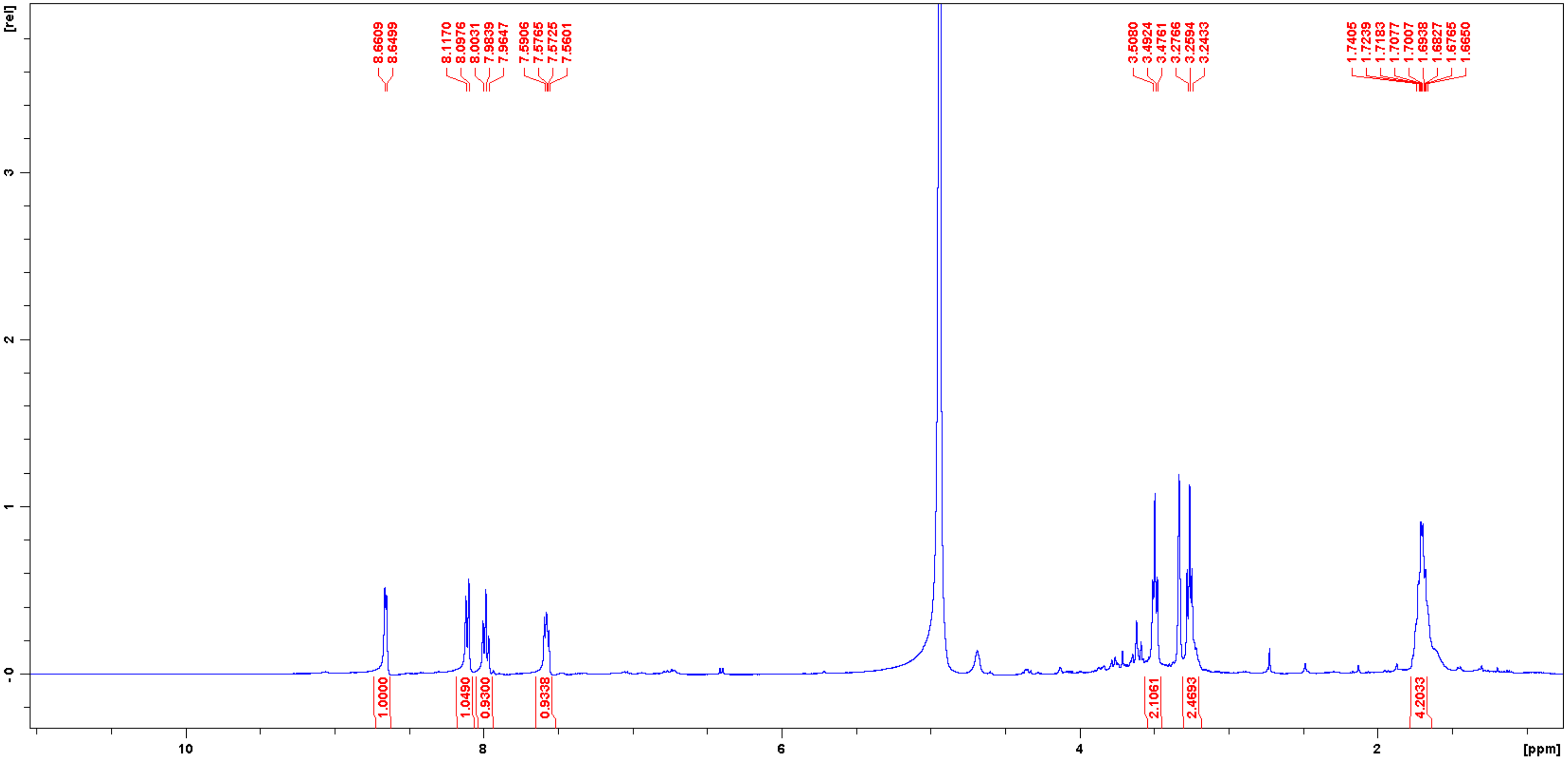

Figure S12. ^{13}C NMR spectrum (CD_3OD) of martensine B (**2**)

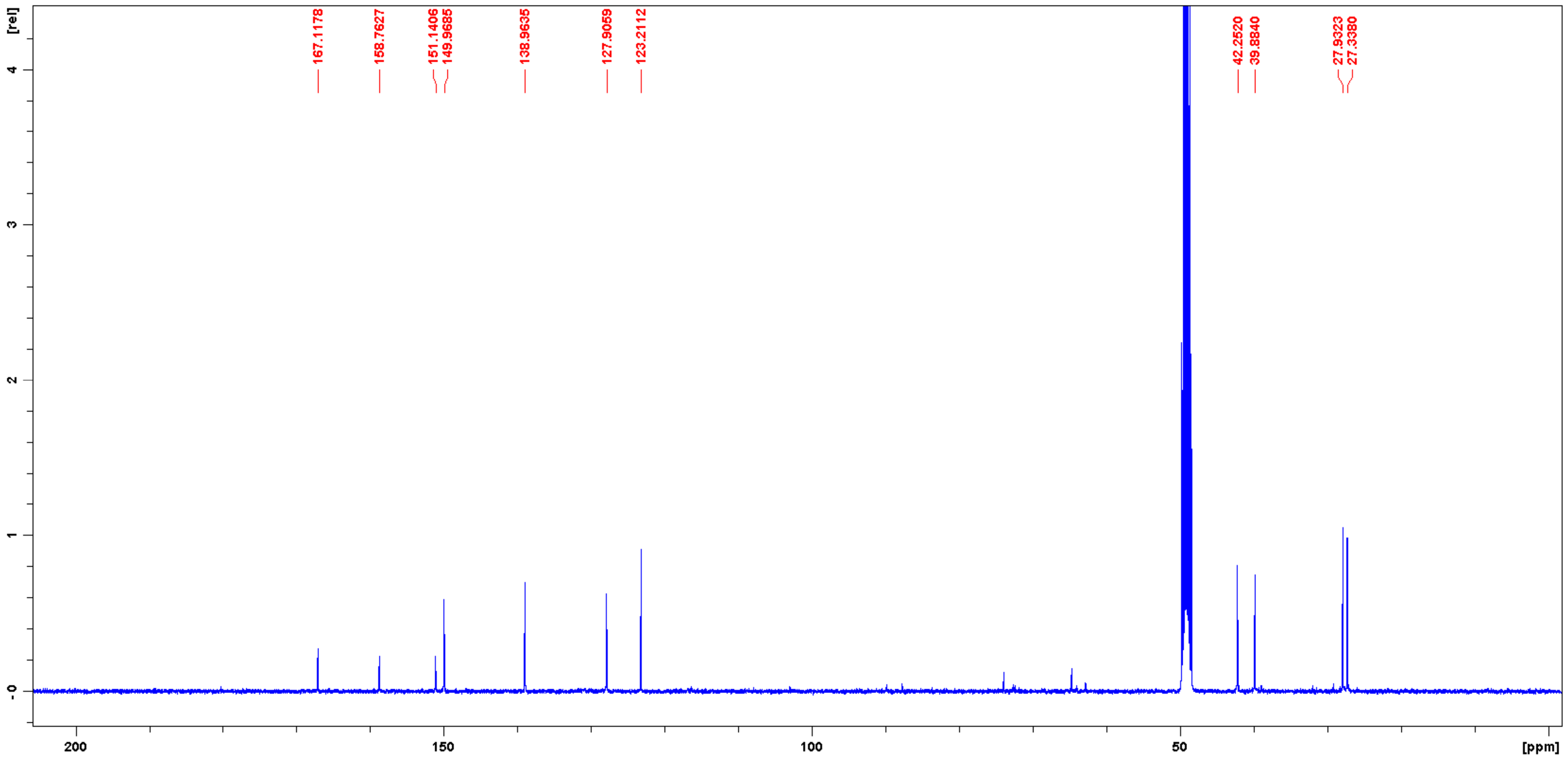

Figure S13. DEPT 135 spectrum of martensine B (**2**)

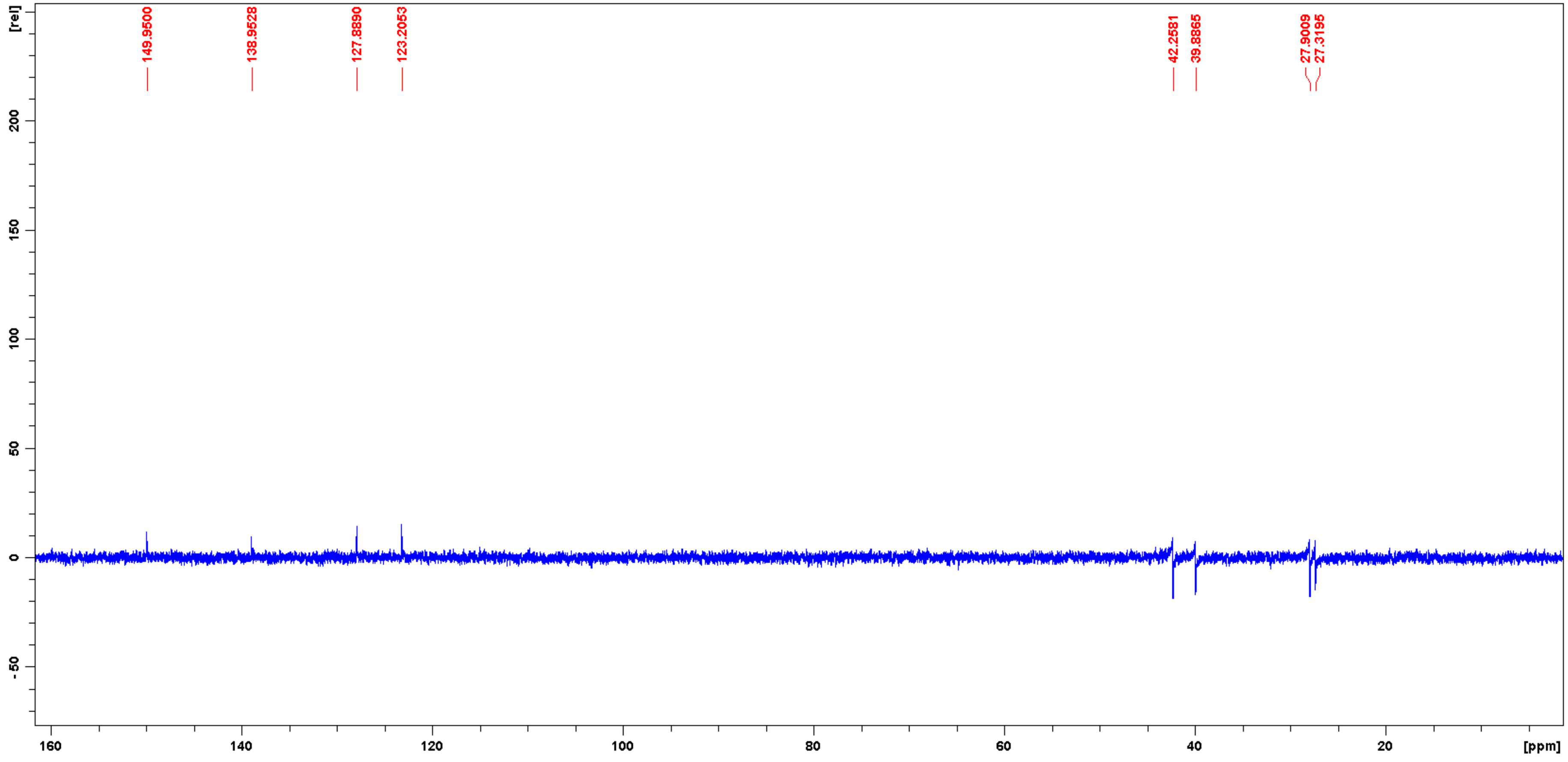

Figure S14. ^{1}H-^{1}H COSY spectrum of martensine B (**2**)

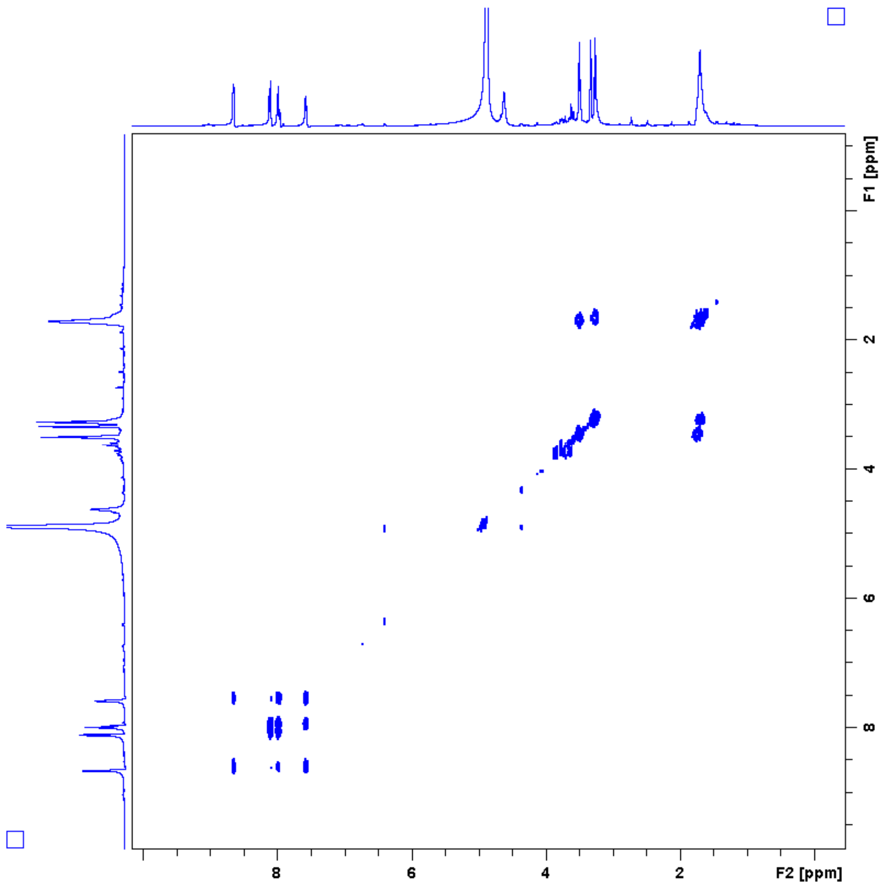

Figure S15. HSQC spectrum of martensine B (**2**)

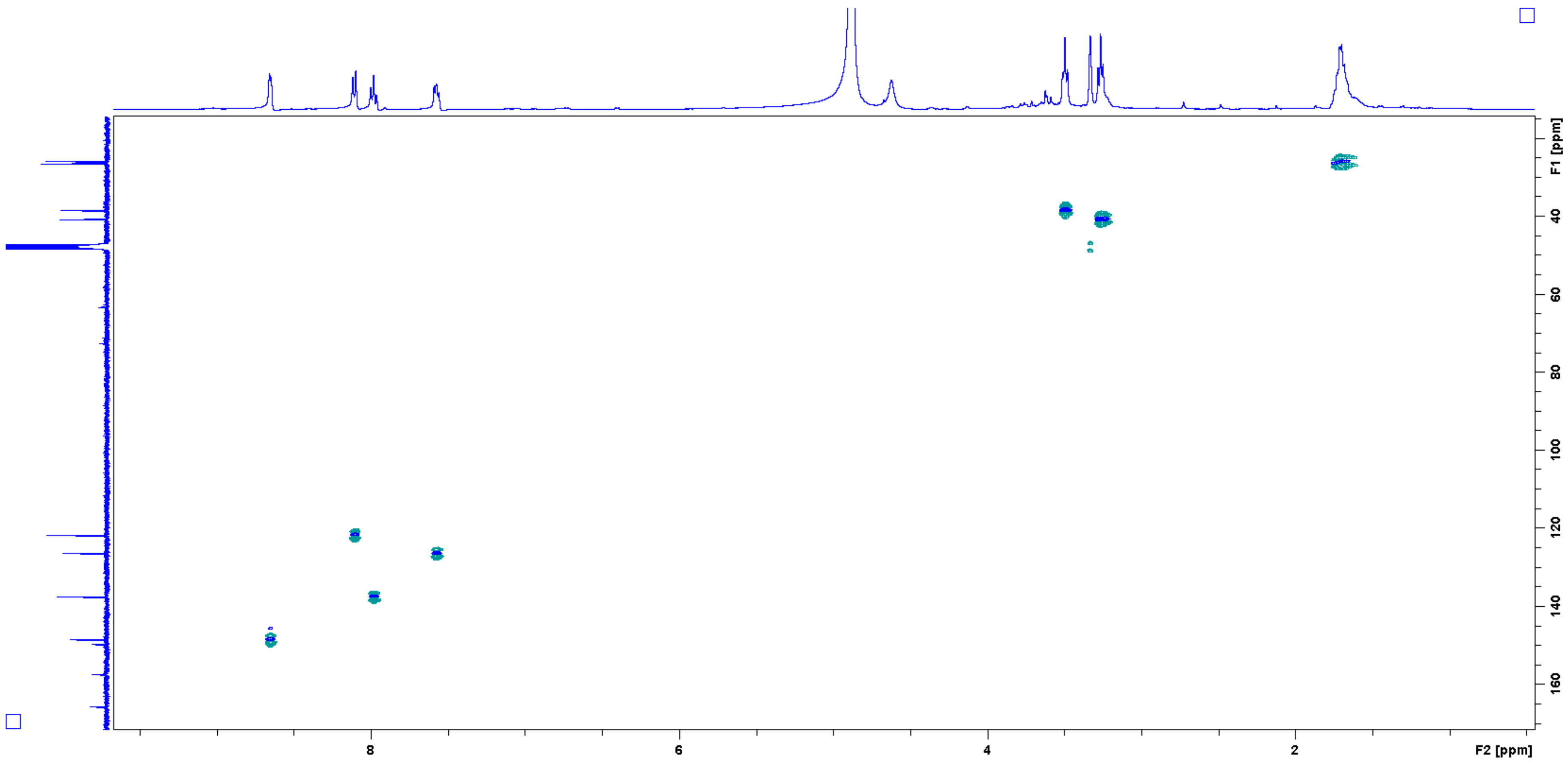

Figure S16. HMBC spectrum of martensine B (**2**)

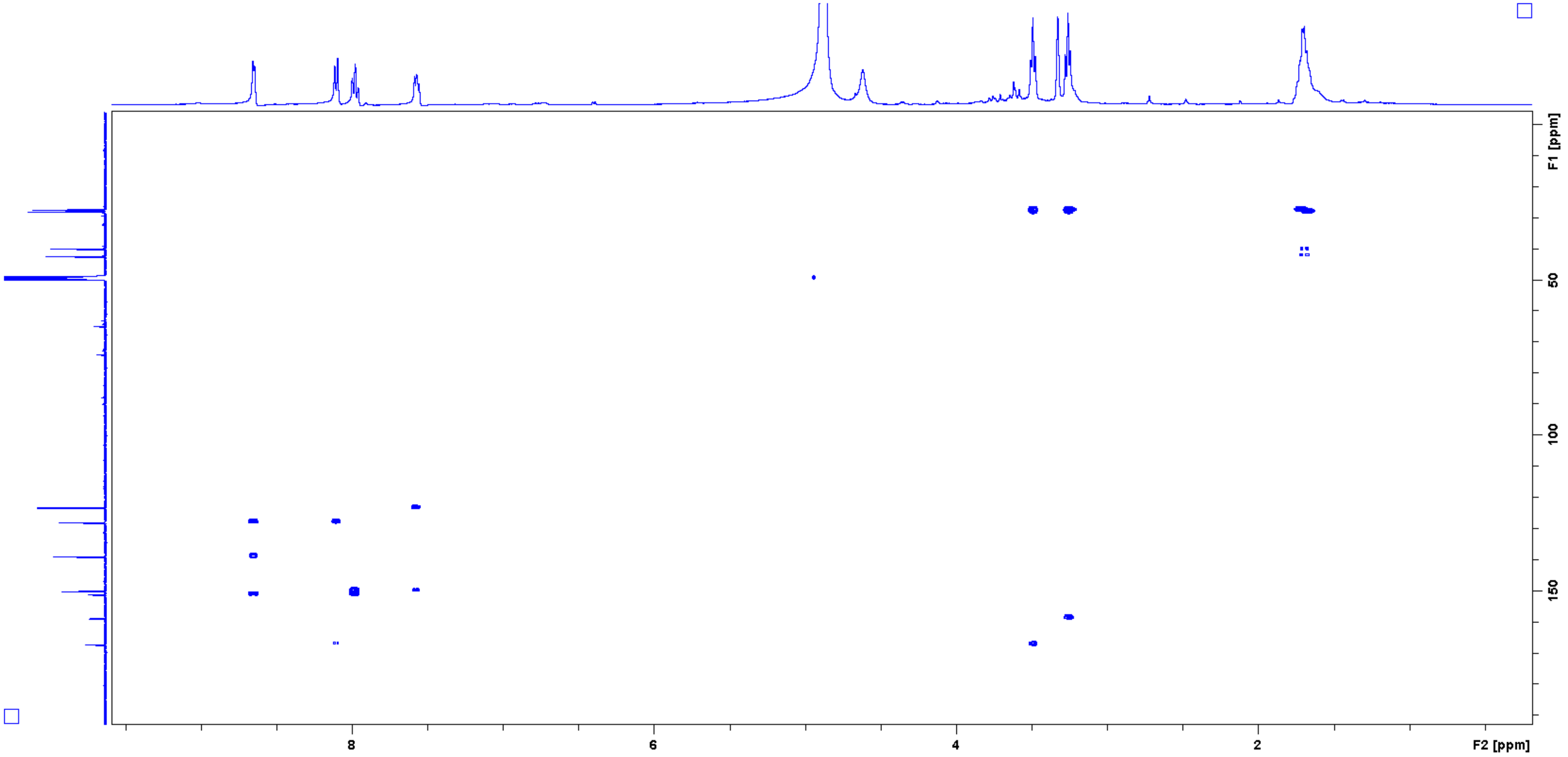

Figure S17. NOESY spectrum of martensine B (**2**)

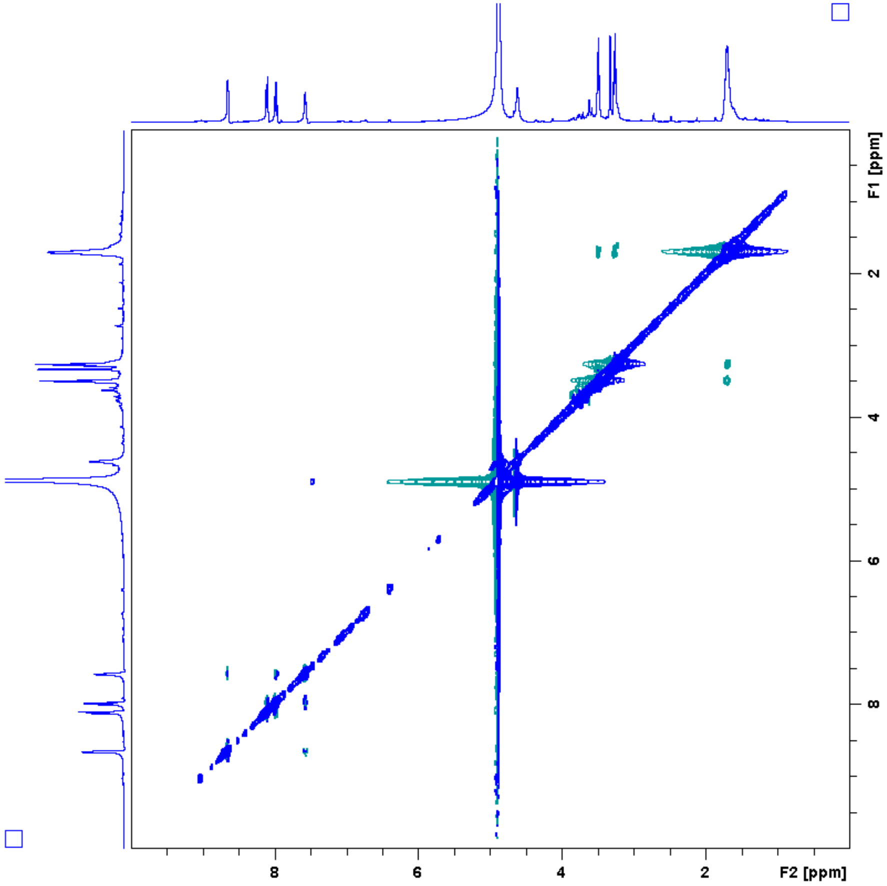

Figure S18. HR-ESI-MS spectrum of trigonelline (**3)**

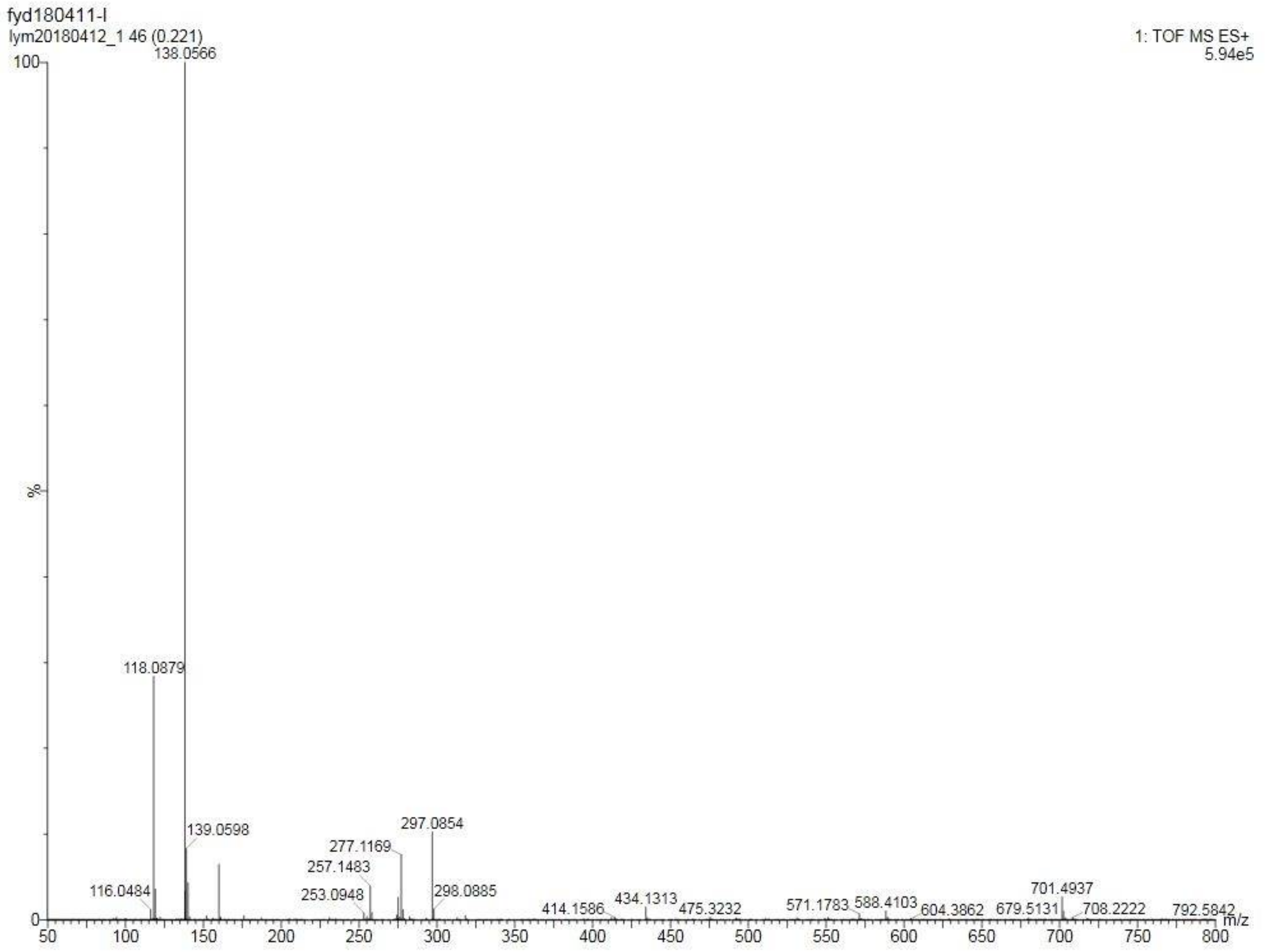

Figure S19. ^{1}H NMR spectrum (CD_3OD) of trigonelline (**3**)

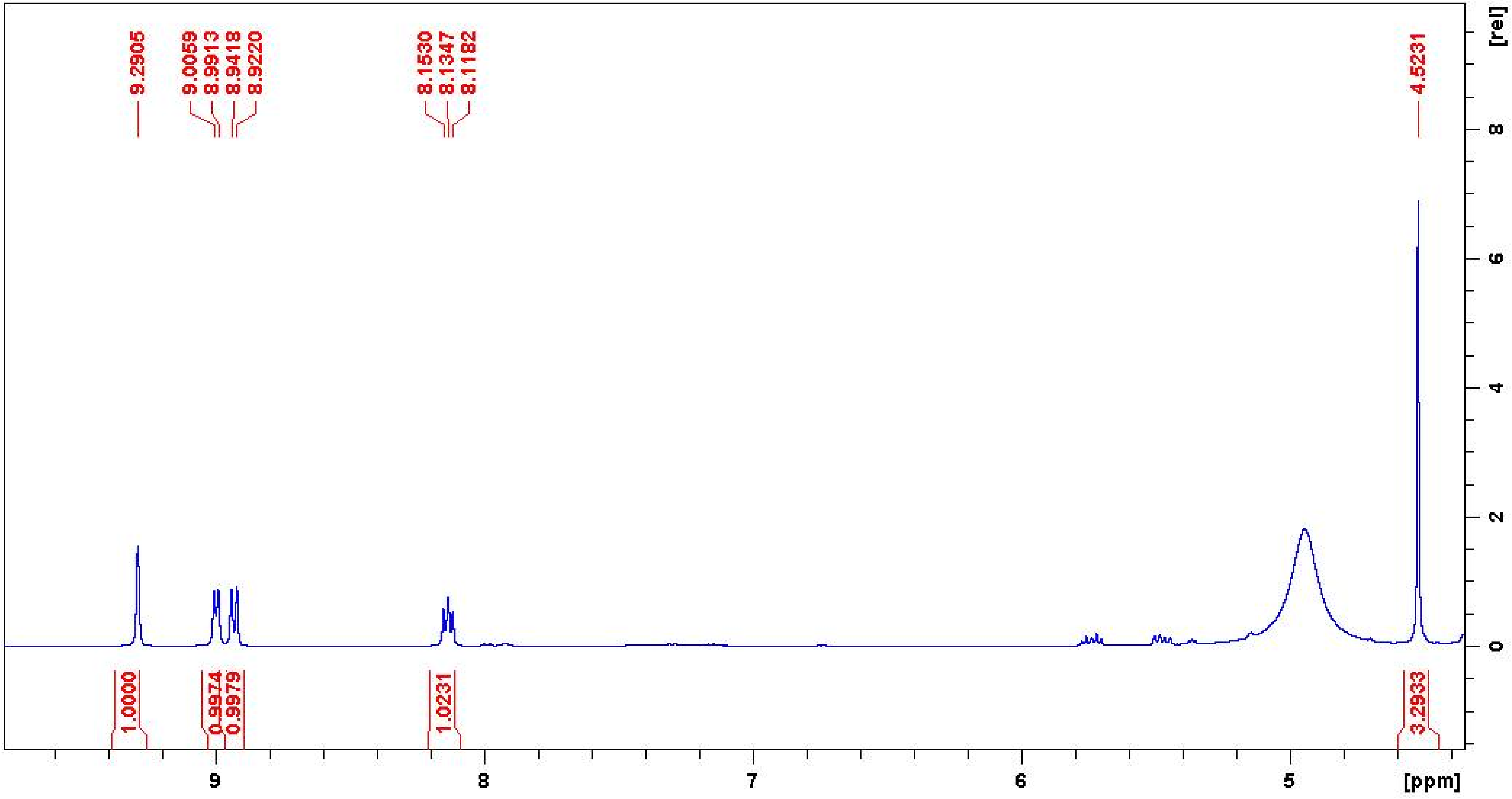

Figure S20. ^{13}C NMR spectrum (CD_3OD) of trigonelline (**3**)

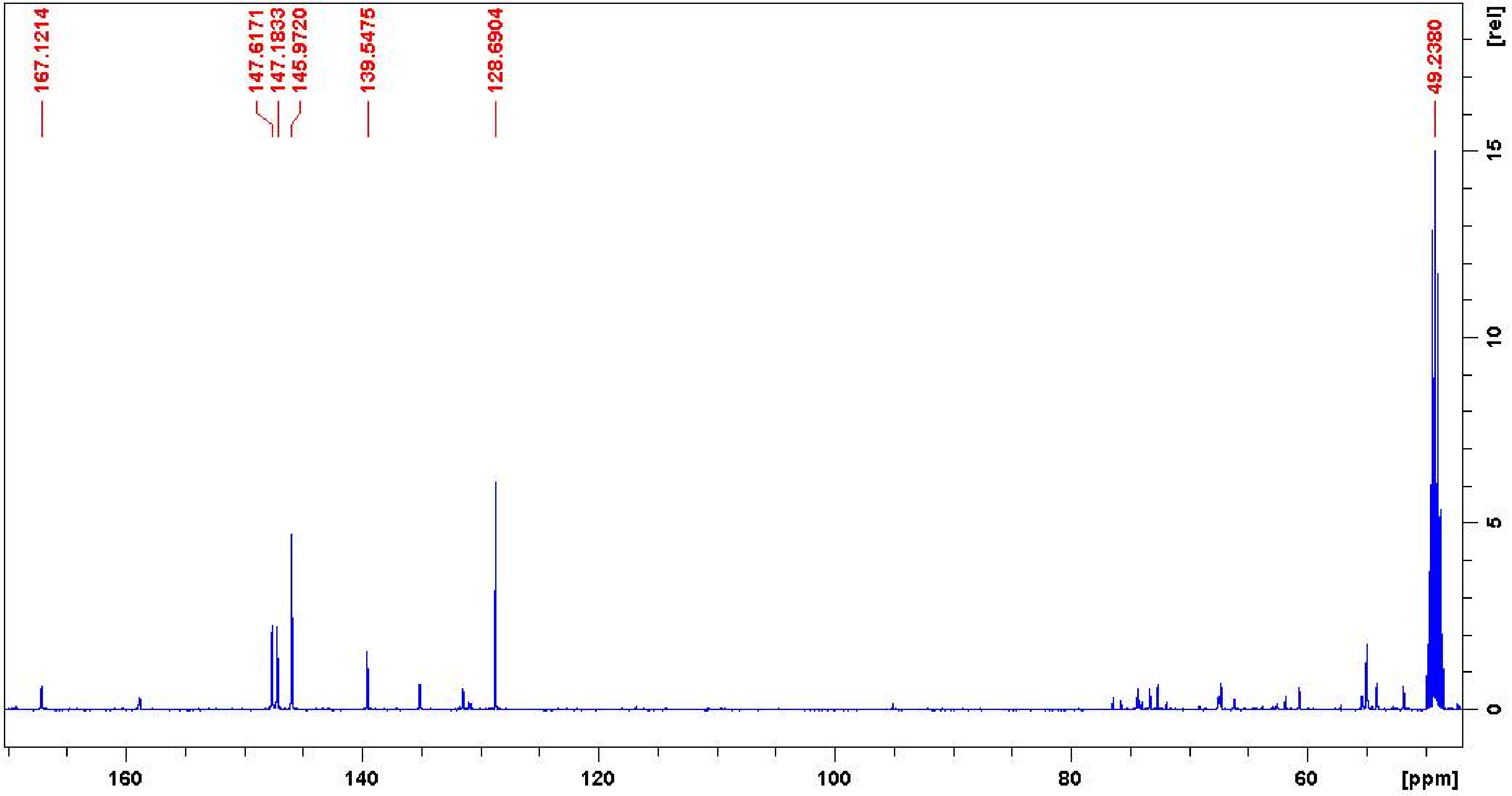

Figure S21. ^{1}H-^{1}H COSY spectrum of trigonelline (**3**)

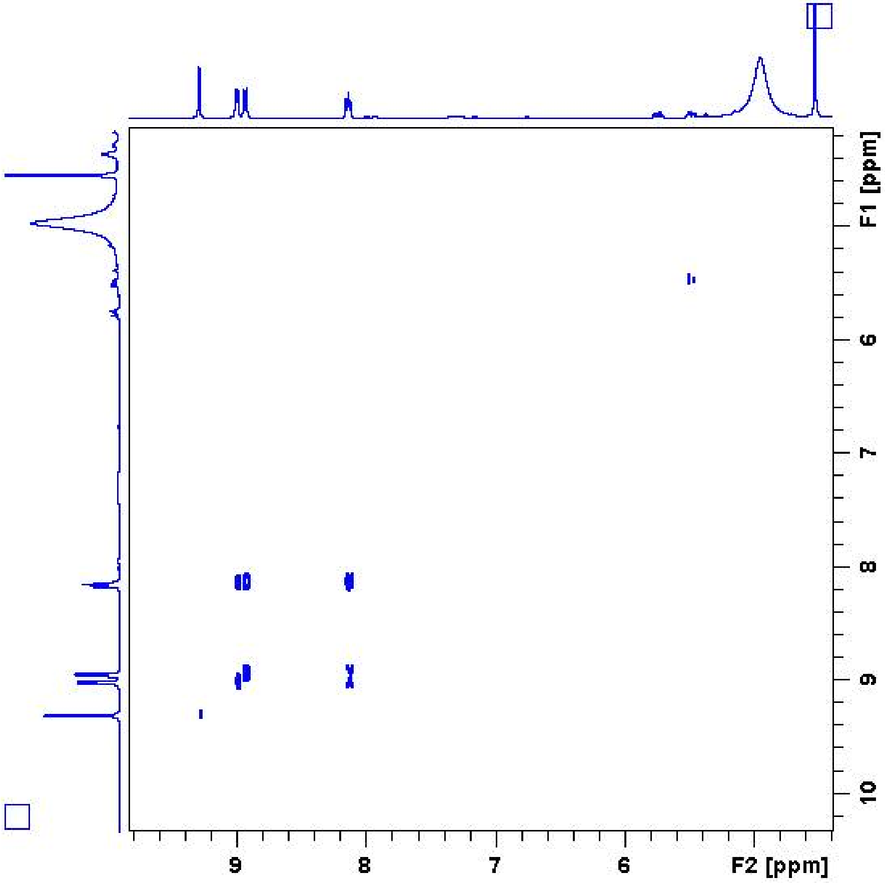

Figure S22. HSQC spectrum of trigonelline (**3**)

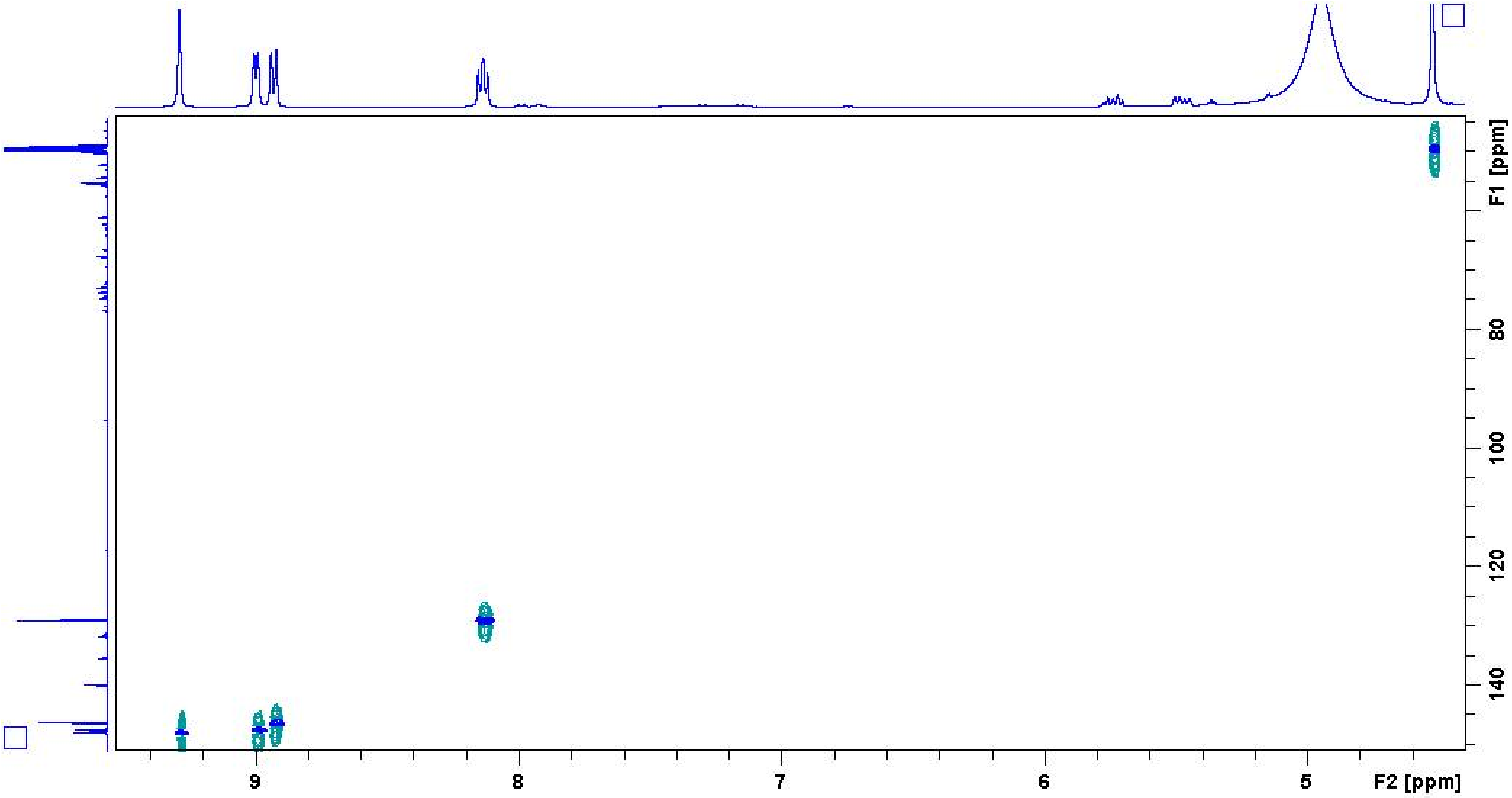

Figure S23. HMBC spectrum of trigonelline (**3**)

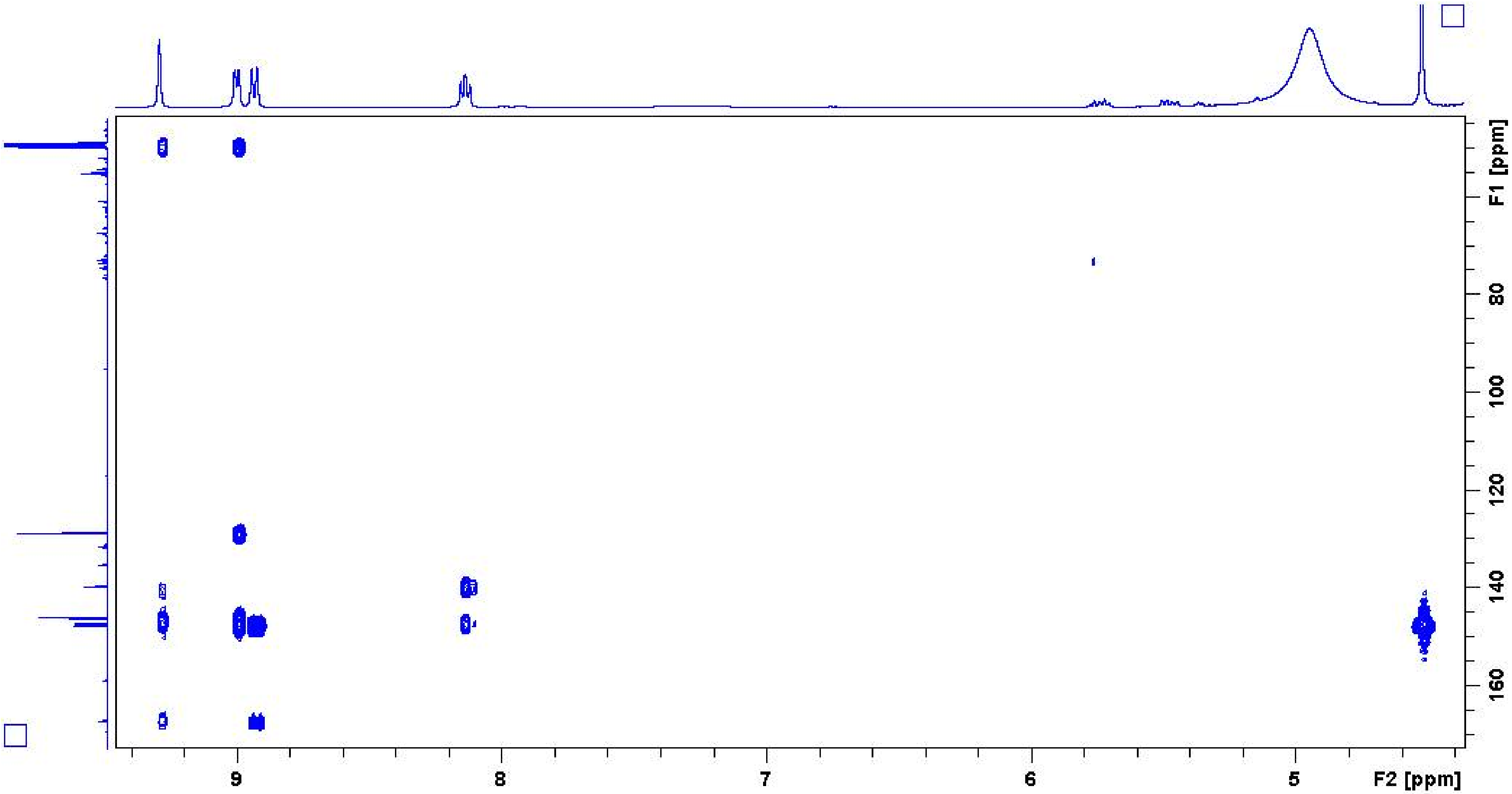

Figure S24. NOESY spectrum of trigonelline (**3**)

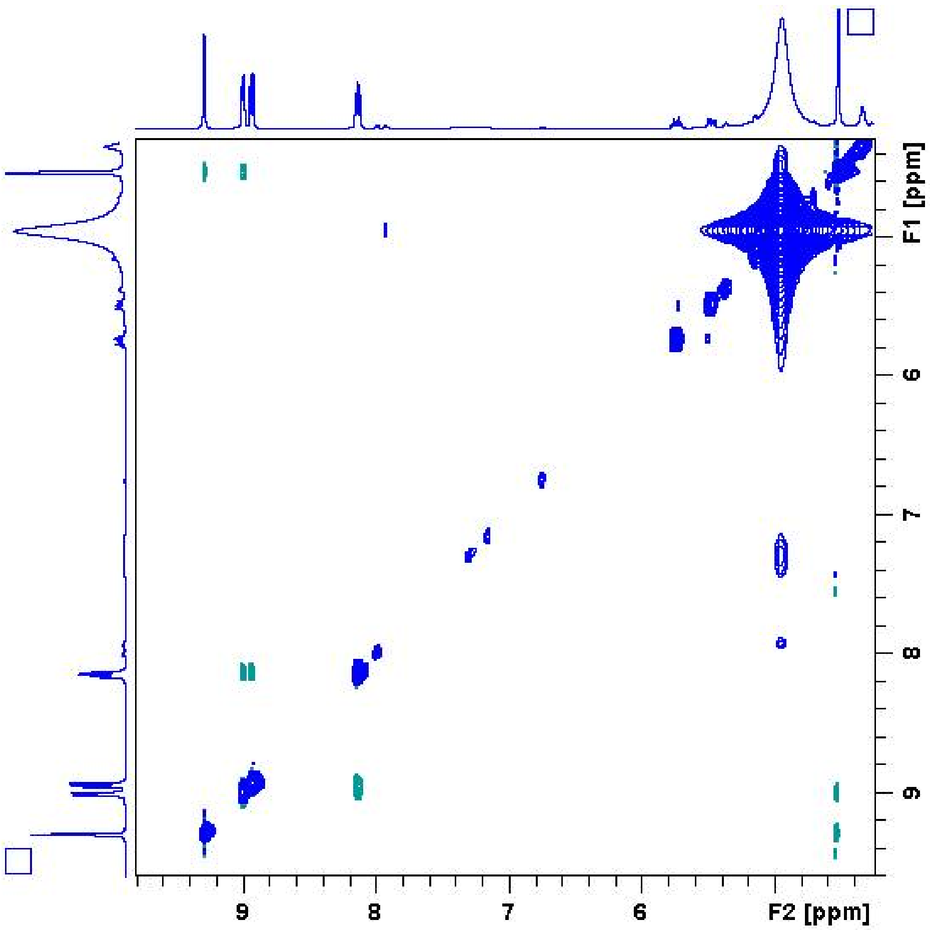

Figure S25. HR-ESI-MS spectrum of 3-methylbuthyl hydrodisulfide (**4**)

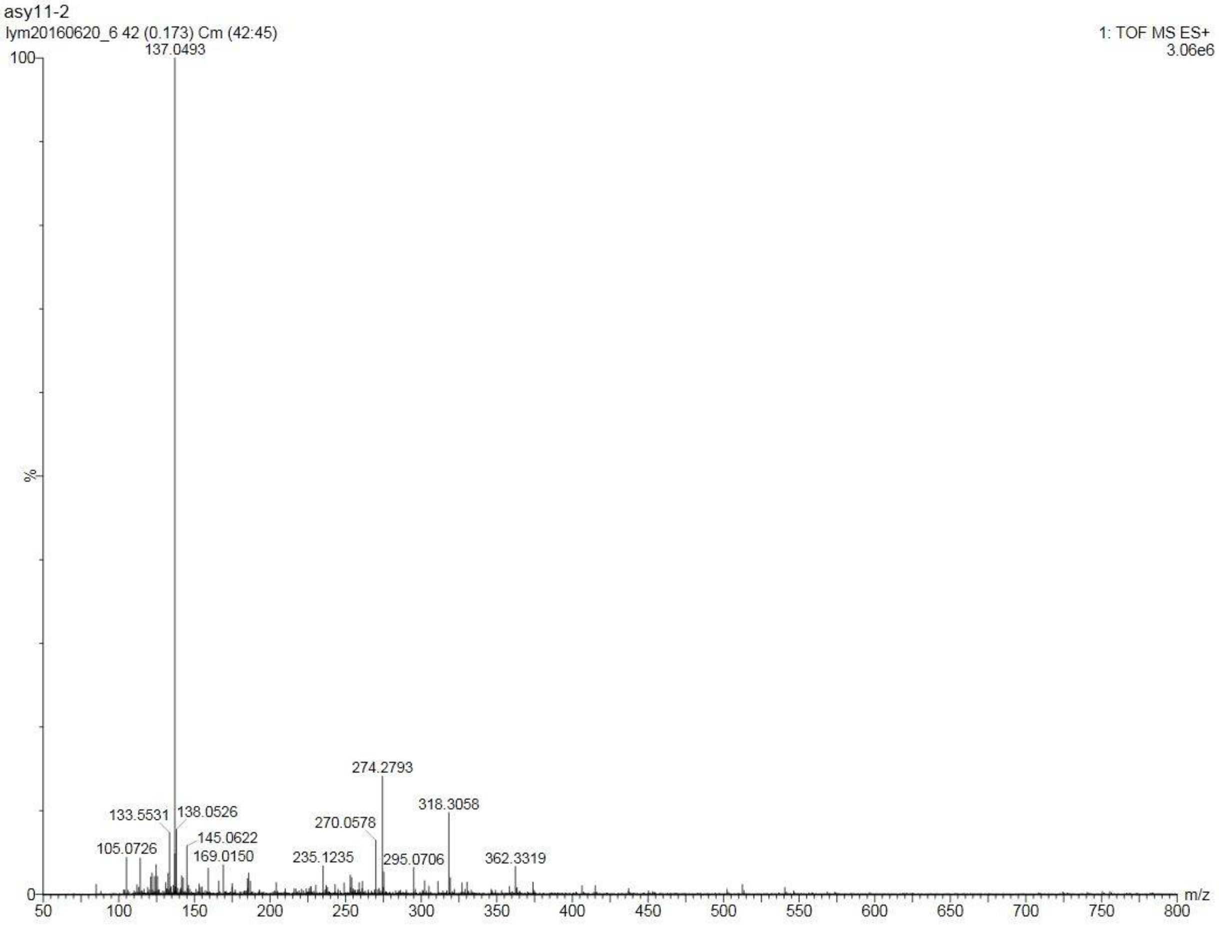

Figure S26. ^{1}H NMR spectrum (DMSO-d_6) of 3-methylbuthyl hydrodisulfide (**4**)

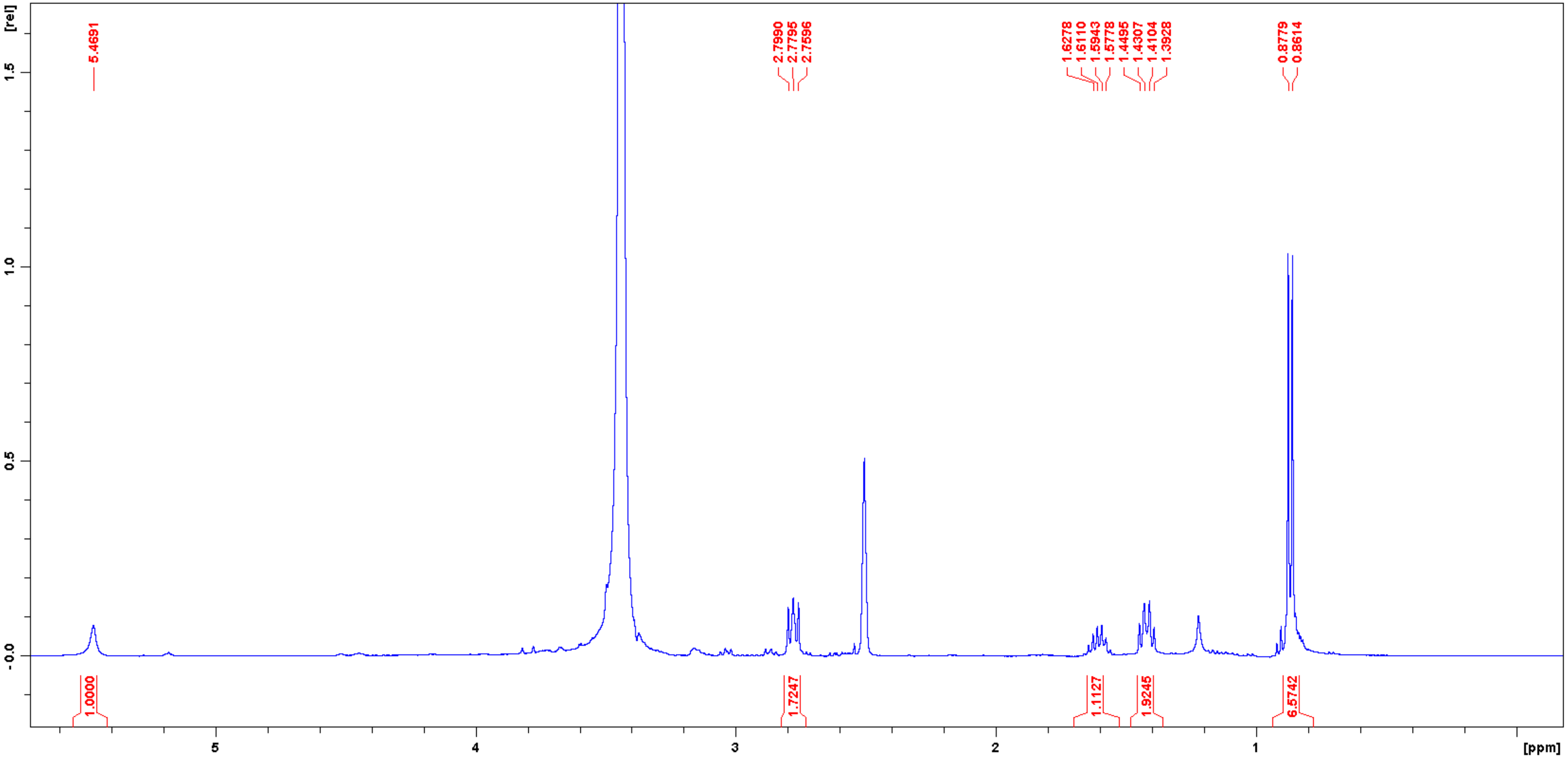

Figure S27. ^{13}C NMR spectrum (DMSO-d_6) of 3-methylbuthyl hydrodisulfide (**4**)

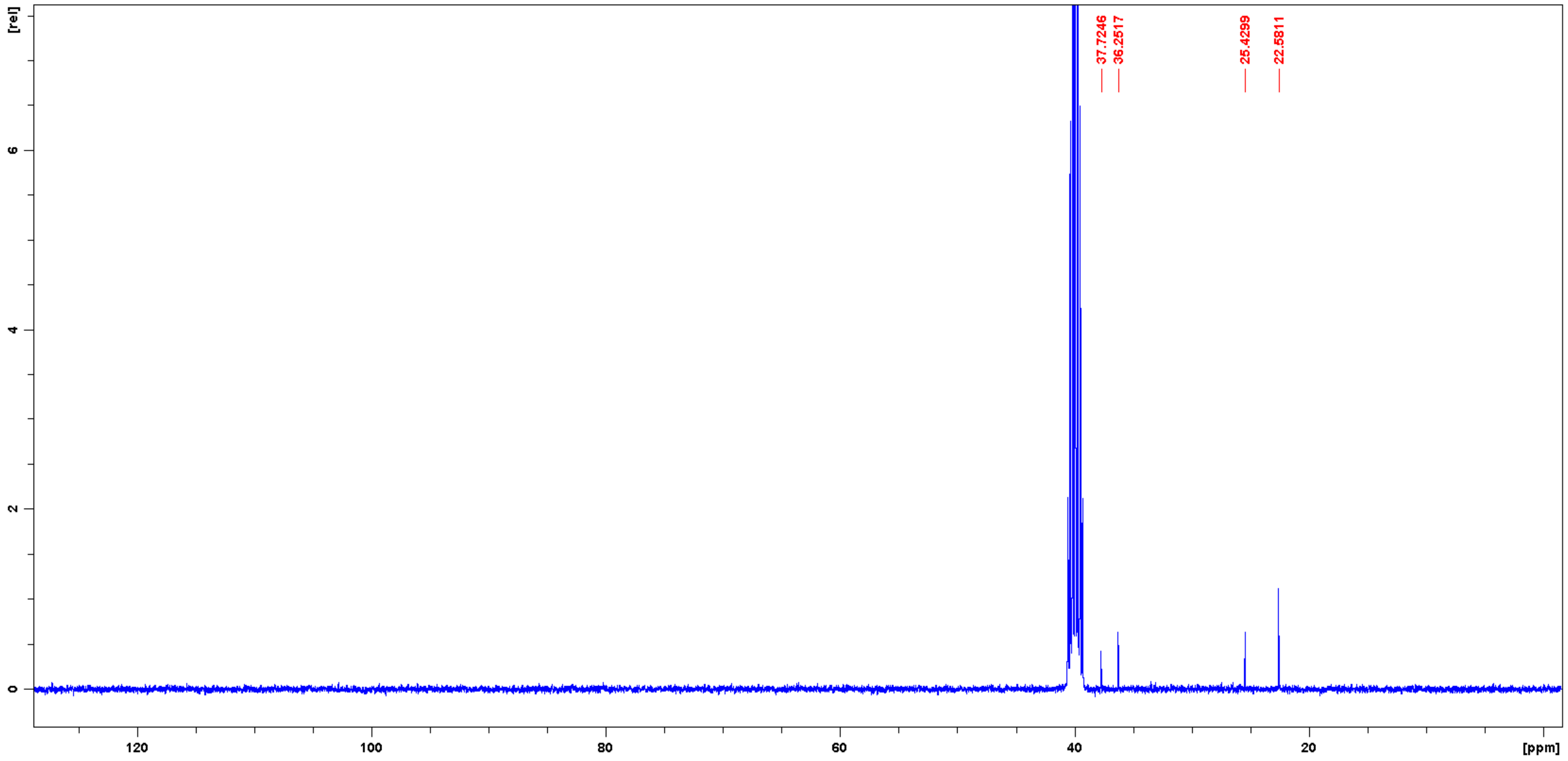

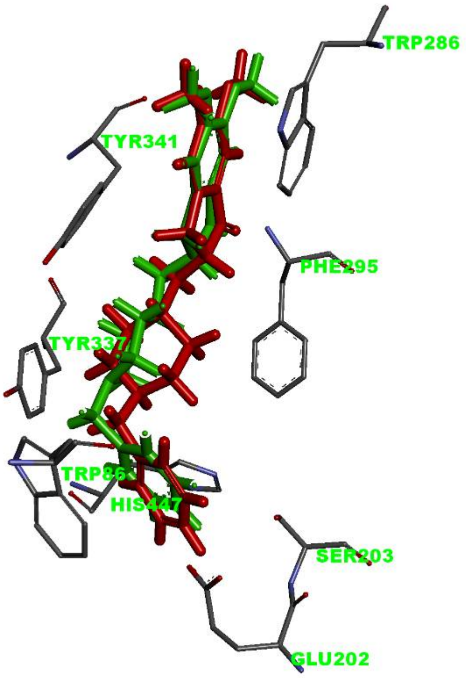

Fig. S28. Docking validation. Redocked donepezil (best-ranked docking solution) is reported in red sticks into the active site of AChE. Crystallographic donepezil is represented as green sticks.

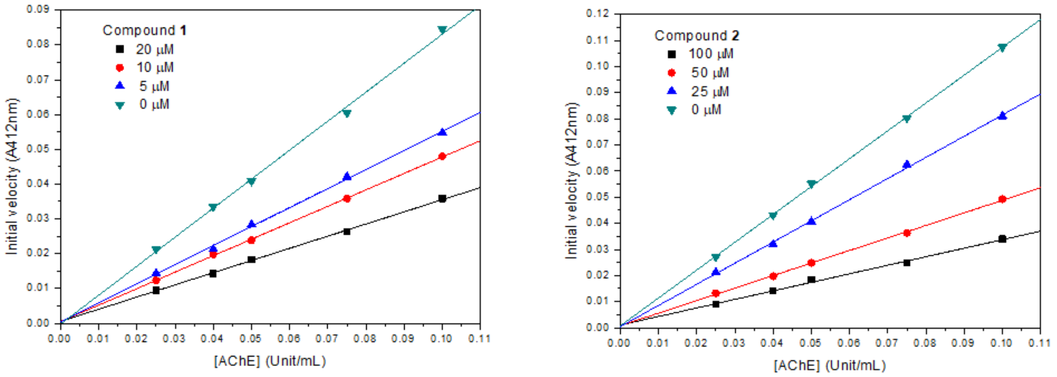

Fig. S29. Plots of initial velocity versus enzyme concentration for the inhibition of compounds **1** and **2** on the hydrolysis activity of AChE.
